# A Critical Review of Short Antimicrobial Peptides from Scorpion Venoms, Their Physicochemical Attributes, and Potential for the Development of New Drugs

**DOI:** 10.1007/s00232-024-00315-2

**Published:** 2024-07-11

**Authors:** Pedro Alejandro Fong-Coronado, Verónica Ramirez, Verónica Quintero-Hernández, Daniel Balleza

**Affiliations:** 1 Ecology and Survival of Microorganisms Group (ESMG), Laboratorio de Ecología Molecular Microbiana (LEMM), Centro de Investigaciones en Ciencias Microbiológicas (CICM), Instituto de Ciencias (IC), Benemérita Universidad Autónoma de Puebla (BUAP), Puebla, México; 2 Facultad de Ciencias Químicas, Benemérita Universidad Autónoma de Puebla (FCQ-BUAP), Ciudad Universitaria, Puebla, México; 3 CONAHCYT-ESMG, LEMM, CICM, IC, BUAP, Ciudad Universitaria, Edificio IC-11, Col. San Manuel, Puebla, México; 4 Laboratorio de Microbiología, Unidad de Investigación y Desarrollo en Alimentos, Instituto Tecnológico de Veracruz, Tecnológico Nacional de México, Veracruz, México

**Keywords:** Scorpion venoms, Short antimicrobial peptides, Hydrophobic moment, Electrostatic potential, Intrinsic flexibility, Lipid packing and curvature

## Abstract

Scorpion venoms have proven to be excellent sources of antimicrobial agents. However, although many of them have been functionally characterized, they remain underutilized as pharmacological agents, despite their evident therapeutic potential. In this review, we discuss the physicochemical properties of short scorpion venom antimicrobial peptides (ssAMPs). Being generally short (13–25 aa) and amidated, their proven antimicrobial activity is generally explained by parameters such as their net charge, the hydrophobic moment, or the degree of helicity. However, for a complete understanding of their biological activities, also considering the properties of the target membranes is of great relevance. Here, with an extensive analysis of the physicochemical, structural, and thermodynamic parameters associated with these biomolecules, we propose a theoretical framework for the rational design of new antimicrobial drugs. Through a comparison of these physicochemical properties with the bioactivity of ssAMPs in pathogenic bacteria such as *Staphylococcus aureus* or *Acinetobacter baumannii*, it is evident that in addition to the net charge, the hydrophobic moment, electrostatic energy, or intrinsic flexibility are determining parameters to understand their performance. Although the correlation between these parameters is very complex, the consensus of our analysis suggests that there is a delicate balance between them and that modifying one affects the rest. Understanding the contribution of lipid composition to their bioactivities is also underestimated, which suggests that for each peptide, there is a physiological context to consider for the rational design of new drugs.

**Graphical Abstract:**

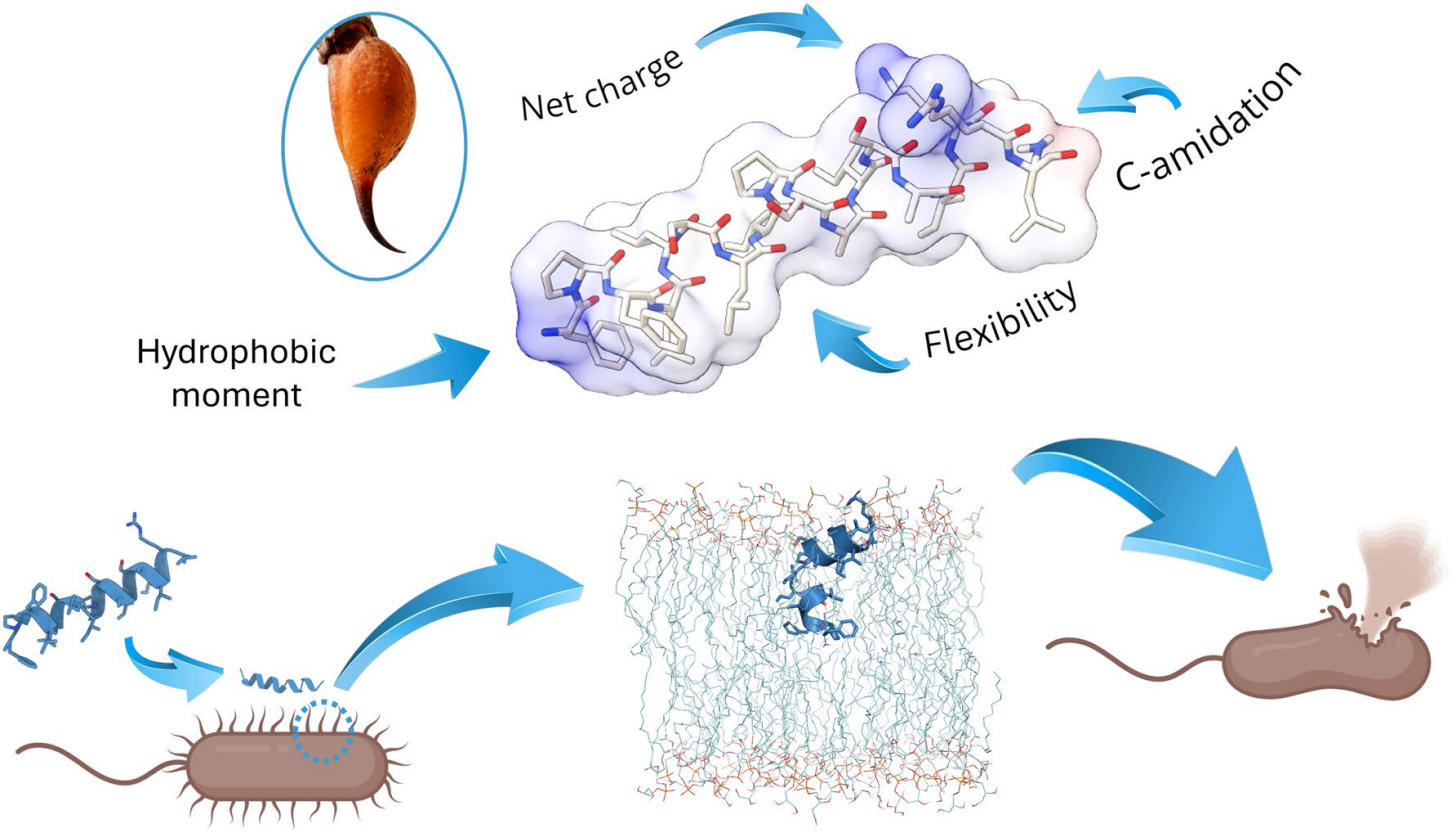

## Introduction

Antimicrobial substances are defined as chemical compounds that cause the death of a microorganism or halt its growth at low concentrations. Antimicrobials are produced by organisms in all three domains of life and they can also be obtained through chemical synthesis or heterologous expression. Besides, it has been proposed that their production is part of the defense mechanism and competition for dominance in ecological niches (Seo et al. 2012; Boparai and Sharma 2019). Despite the diversity of known antimicrobial substances, resistance to these compounds has also evolved, representing one of the greatest threats to human health, animal health, and agriculture. Antimicrobial resistance (AMR) arises when microorganisms develop the ability to resist its deleterious effects, which reduces the efficacy of drugs designed to combat infectious diseases. Several factors contribute to the increase in AMR, one of which is the indiscriminate use of broad-spectrum antibiotics, since through “selective pressure” phenomena, the development of microorganisms resistant to these compounds is facilitated, which in turn reduces the number of sensitive microorganisms.

Today, microorganisms are known to be resistant to multiple antimicrobials, and those microorganisms that are resistant to three or more different classes of antimicrobials are known as multidrug-resistant (MDR) (Santajit and Indrawattana 2016). Recently, the World Health Organization (WHO) published a list of MDR microorganisms for which new antibiotics need to be developed; they include *Enterococcus* spp., *Staphylococcus aureus*, *Klebsiella pneumoniae*, *Acinetobacter baumannii*, *Pseudomonas aeruginosa*, and *Enterobacter* spp. Another microorganism of global clinical importance is *Mycobacterium tuberculosis*, which causes the death of millions of people each year due to the increase in MDR strains; in addition, extreme drug-resistant tuberculosis (XDR-TB) infections have been reported worldwide (WHO 2023).

Due to the high incidence of MDR microorganisms in different sectors worldwide and the limitation to effective antimicrobials against these pathogens, AMR is considered a global threat and a critical problem in terms of human health since the risk of spread and persistence of infectious diseases is increasing. Therefore, it is imperative to seek new strategies for the control of MDR microorganisms, which makes the study of new antimicrobial substances pivotal to determine their biological activity and select those with the potential to design and develop new and more effective drugs. An alternative is to use peptides with antimicrobial activity (AMPs) which are synthesized particularly by different amphibia and venomous animals (Primon-Barros and José Macedo 2017; Patocka et al. 2019).

Magainins, initially discovered in the African frog skin *Xenopus laevis*, show antimicrobial activity against a broad spectrum of microorganisms, including Gram-positive and Gram-negative bacteria, fungi, and also certain viruses. These AMPs have been central in drug design research, not only for their therapeutic potential but also for helping to better understand how natural organisms defend themselves against microbial infections (Patocka et al. 2019). On the other hand, unlike the AMPs discovered in amphibians, those present in the venoms of certain species of snakes have more recently also been positioned as excellent candidates for the development of new drugs against multidrug-resistant human pathogens and for the treatment of cancer (Pérez-Peinado et al. 2020). Scorpion venom is another source of AMPs and also contains a wide variety of biochemical compounds, among which are several toxins that modulate the function of ion channels and receptors in the membranes of excitable cells, and which are responsible for the multiple known symptoms of poisoning (Quintero-Hernández et al. 2013). Bioactive AMPs against bacteria, fungi, yeasts, and viruses have also been isolated from scorpion venoms (Harrison et al. 2014; Rincón-Cortés et al. 2022). However, only a few scorpion AMPs have been evaluated against multidrug-resistant bacterial strains (Al-Asmari et al. 2017; Cesa-Luna et al. 2019). Even so, AMPs found in scorpion venoms stand as potential therapeutic agents to be deeply characterized and used in the design and development of new-generation antimicrobial drugs.

Scorpion AMPs, present in the venom gland, could have a protective role against pathogenic infections or potentiate toxin effects. They have been classified into several groups, which include Cys-containing scorpines, long-chain non-Cys-containing AMPs, and short-chain non-Cys-containing AMPs (Harrison et al. 2014). Short antimicrobial peptides present in scorpion venoms (ssAMPs) are composed of about 13 to 20 amino acid residues, they lack disulfide bridges and are generally amidated at the last amino acid of the C-terminal region. The first characterized peptide of this type was named IsCT from the venom of the scorpion *Opisthacanthus madagascariensis*. This is a 13-aa residue peptide (ILGKIWEGIKSLF-NH_**2**_) and its characterization exhibited low hemolytic activity and high antibacterial activity against Gram-positive and Gram-negative bacteria, which immediately indicated its high therapeutic potential (Dai et al. 2001, 2002).

**Fig. 1:**
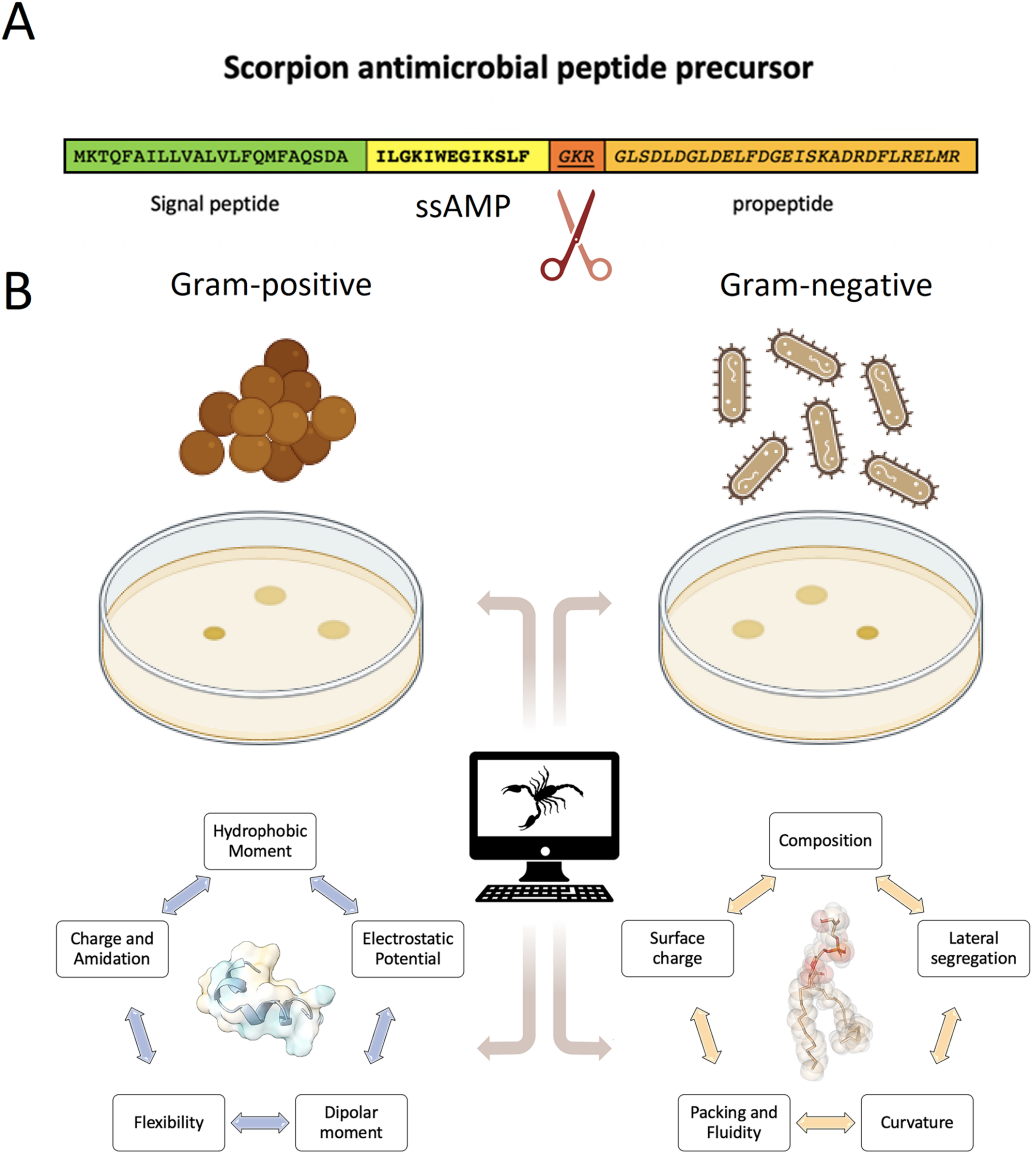
Enzymatic processing of amidated scorpion peptides and main physicochemical properties contributing to their bioactivity. **A** The precursor of ssAMPs consist of a signal peptide at the N-terminal, followed by the sequence of the mature peptide with the antimicrobial property, a ‘GKR’ motif is enzymatically recognized and exscinded, while the C-terminal propeptide is released. **B** The activity of mature peptides against Gram-positive and Gram-negative bacteria depends on their physicochemical properties, which can be optimized in silico, as well as a function of the lipid properties

The precursors of these biomolecules consist of a signal sequence, a mature peptide that codes the antimicrobial activity, and a propeptide (Fig. 1A). The precursor undergoes a post-translational modification to release the ssAMP. The IsCT precursor comprises 68 amino acids, but the active peptide corresponds to amino acids 24 to 36, while the propeptide spans residues 37 to 68. A 3-aa proteolysis motif, GKR or GRR, is located between the active sequence and the propeptide, which are recognition sites for subsequent post-translational enzymatic modifications (Delgado-Prudencio et al. 2019, 2022). At this site, a cleavage occurs and, simultaneously, the C-terminal hydroxyl group of carbon-1 in the last residue is replaced by an amide group, producing the mature peptide. Amidation at the C-terminus of ssAMPs seems to be crucial for the activity of these bioactive agents since it has been experimentally proven that the removal of this chemical modification eliminates the antimicrobial activity of ssAMPs from the venom of the Australian scorpion *Urodacus yaschenkoi* (Luna-Ramirez et al. 2013).

**Fig. 2:**
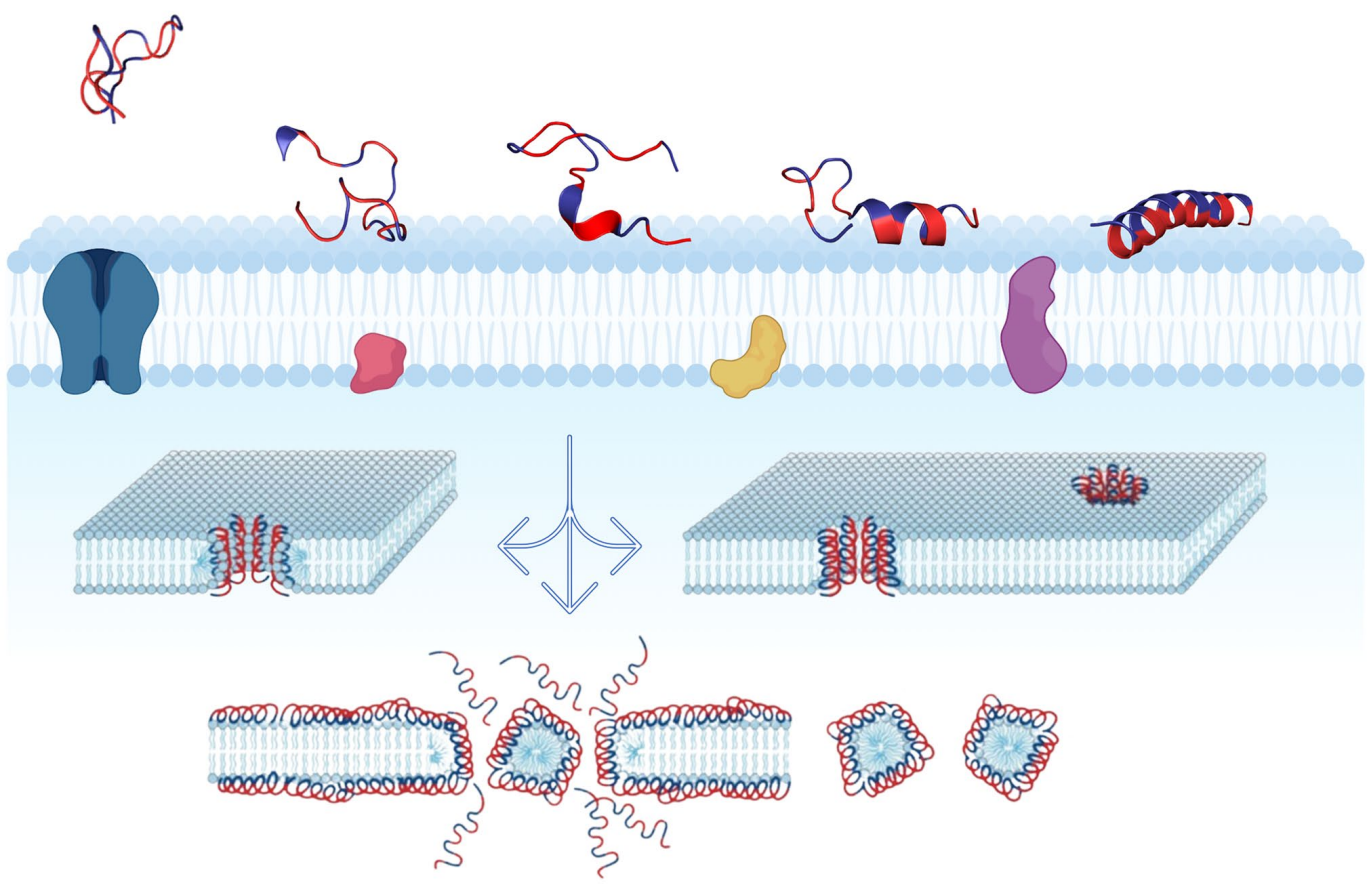
Membrane permeation by pore formation or the carpet model. Cationic antimicrobial peptides interact with surface negative charges on the surface of biomembranes. During this interaction, the peptides transit to ordered α-helical states that can destabilize the integrity of the lipid bilayer through the formation of toroidal pores (*left*), barrel pores (*right*) or micellization processes coupled to a carpet effect (*bottom*)

To date, 134 ssAMPs, composed of less than 20-aa residues, have been reported in the Database of Antimicrobial Activity and Structure of Peptides, DBAASP (https://dbaasp.org). Although the mechanism of action of most of these ssAMPs still is unknown, it has been proposed that they can form membrane pores and cell lysis as has been proposed for magainins, for example (Harrison et al. 2014). According to the standard model, the amphipathic nature of AMPs allows them to be inserted into the lipid membrane in close dependence on the physicochemical properties of these biomolecules. In that sense, the electrostatic interactions between AMPs and the surface of the lipid membrane can evolve to form pores through hydrophobic interactions by concentration-dependent oligomerization mechanisms and eventually solubilize the membrane (Lee et al. 2016; Juhl et al. 2021) (Fig. 2). Other possible mechanisms of action that explain the effects of AMPs include their role in interfering with protein synthesis, cell wall synthesis, DNA damage, and autolysis induction (Nicolas 2009). On the other hand, it is well known that the bioactivity of AMPs and their potential for the development of new drugs is directly associated with lipid composition (Lee et al. 2016; Giuliani et al. 2008). The membranes of animal cells are rich in neutral phospholipids and cholesterol, which inhibit the incorporation of these peptides into the membrane and the subsequent formation of aqueous pores. In that sense, the concentrations to damage the membranes of eukaryotic cells are significantly higher compared to those necessary to inhibit bacteria, whose composition is rich in anionic lipids (van Voorst and de Kruijff 2000). This represents an advantage for the therapeutic use of AMPs and ssAMPs in particular.

Thus, the development of new drugs is directly associated with exploiting the great potential that ssAMPs have. Given its proven bioactivity and the fact that being so small, their chemical synthesis is facilitated, with this review, we intend to offer for the first time a very extensive vision of the properties that these short peptides possess, the lipids with which they interact and how such properties can be modeled with algorithms based on artificial intelligence to explore and design new, increasingly efficient antimicrobial drugs (Fig. 1B). Finally, we briefly discuss some studies where certain ssAMPs are already being evaluated as potential new drugs.

## Physicochemical Parameters Determining the Lytic Activity of ssAMPs in Lipid Bilayers

From a functional context, trying to elucidate the actions exerted by these ssAMPs in lipid biomembranes and model systems will allow us to establish the theoretical-conceptual framework for the rational design of peptides with therapeutic potential. In this section, nine key parameters which depend on the amino acid composition of ssAMPs are included: (1) hydrophobicity, (2) amphipathicity, (3) the hydrophobic moment, (4) net charge, (5) the electrostatic potential, (6) dipolar moment, (7) helicity, (8) intrinsic flexibility, and (9) C-terminal amidation.

### Hydrophobicity (H)

The term “hydrophobic” refers to a phenomenon of molecular solvation related to entropy. Hydrocarbons and a large number of nonpolar compounds have poor solubilities in water but dissolve well in organic solvents. From a thermodynamic perspective of the peptide folding, this has important energetic implications. The energy associated with folding as an effect of the water repulsion of hydrophobic amino acids in a polypeptide chain is known as ‘hydrophobic factor’ (Baldwin 2007). One way to quantify this effect is to describe in enthalpic and entropic terms the preference of nonpolar molecular surfaces to interact with other nonpolar surfaces, thus displacing water molecules during the interaction (Motiejunas and Wade 2007).

The relationship between hydrophobicity and antimicrobial activity has been extensively studied since the discovery of animal AMPs. Indeed, a correlation between hydrophobicity of the nonpolar face of amphipathic helices with peptide helicity and bioactivity has been reported (Chen et al. 2007). Furthermore, it has been proven that the presence of hydrophobic residues with bulky side chains (*v. gr.* Trp) increases the hydrophobic surface of the peptides, which contributes to greater penetration into the acylated core of the lipid membranes and increases their antibacterial activity (Yu et al. 2013). In Ponericin L1, for example, with antibacterial activity discovered in the ant *Neoponera goeldii*, it was demonstrated that the binding activity to lipid vesicles depends on the relative hydrophobicity of the typically hydrophobic positions in the natural peptide (Schifano and Caputo 2022). Given that there is a well-established scale of degrees of hydrophobicity and therefore each amino acid can be identified based on its water solubility (Kyte and Doolittle 1982), these results are not surprising but are of great relevance for the rational design of AMPs. Consistent with these data, short melittin/cecropin A hybrid peptides (14–16 aa) were previously described and it was reported that their bioactivities against *S. aureus* can be modulated by subtly modifying the hydrophobicity of the middle residue at position 8 of those peptides (Juvvadi et al. 1996).

These subtle effects of hydrophobicity have also been reported in two ssAMPs members of the Pantinin-like group. In peptides derived from BmKn1 (FIGAVAGLLSKIF-NH_**2**_) and BmKn2 (FIGAIARLLSKIF-NH_**2**_), discovered in the venom of the scorpion *Buthus martensii*, de la Salud Bea et al. (2015a, b) demonstrated that by substituting hydrophobic residues in specific positions with Ala, Val, and Leu on the hydrophobic face, the antimicrobial activity is considerably increased and the percentage of hemolysis is reduced. Similarly, substitutions with the same amino acids in analogous positions of the peptides IsCT1 (ILGKIWEGIKSLF-NH_**2**_) and IsCT2 (IFGAIWNGIKSLF-NH_**2**_) present in the venom of *Opithancatus madagascariensis* reduce its hemolytic activity (de la Salud et al. 2017).

On the other hand, when mutants with lower hydrophobicity were tested in peptides derived from VmCT1 (FLGALWNVAKSVF-NH_**2**_) present in the venom of the endemic central Mexican brown scorpion *Vaejovis mexicanus*, the effect of replacing an Ala residue in position 9 with a Trp—peptide [W]9-VmCT1-NH_**2**_—slightly improves antimicrobial activity against Gram-positive and Gram-negative ATCC bacteria, including *Serratia marcescens*, *E. cloacae*, *S. aureus*, and *B. subtilis*, but without major effect on fungal pathogens of the genus *Candida* and *Aspergillus* (Pedron et al. 2017). This could indicate that the presence of the electron-rich indole group present in the Trp side chain acts to lower the typical hydrophobicity of the methyl group of the original Ala residue.

In sum, a slight but noticeable relationship can be established between increasing the degree of hydrophobicity in specific positions and enhancing the antimicrobial activity of the ssAMPs. However, this relative influence of hydrophobicity of the ssAMPs mainly affects the hemolytic effect, instead of the antibacterial effect (Dathe and Wieprecht 1999). All the evidence reviewed here indicates, however, that not only the hydrophobicity per se is determinant, but the degree of amphipathicity displayed by these peptides could be the key parameter. This physicochemical factor hand in hand with the hydrophobic moment will be exposed and discussed in the following sections.

### Amphipathicity (Φ)

**Fig. 3:**
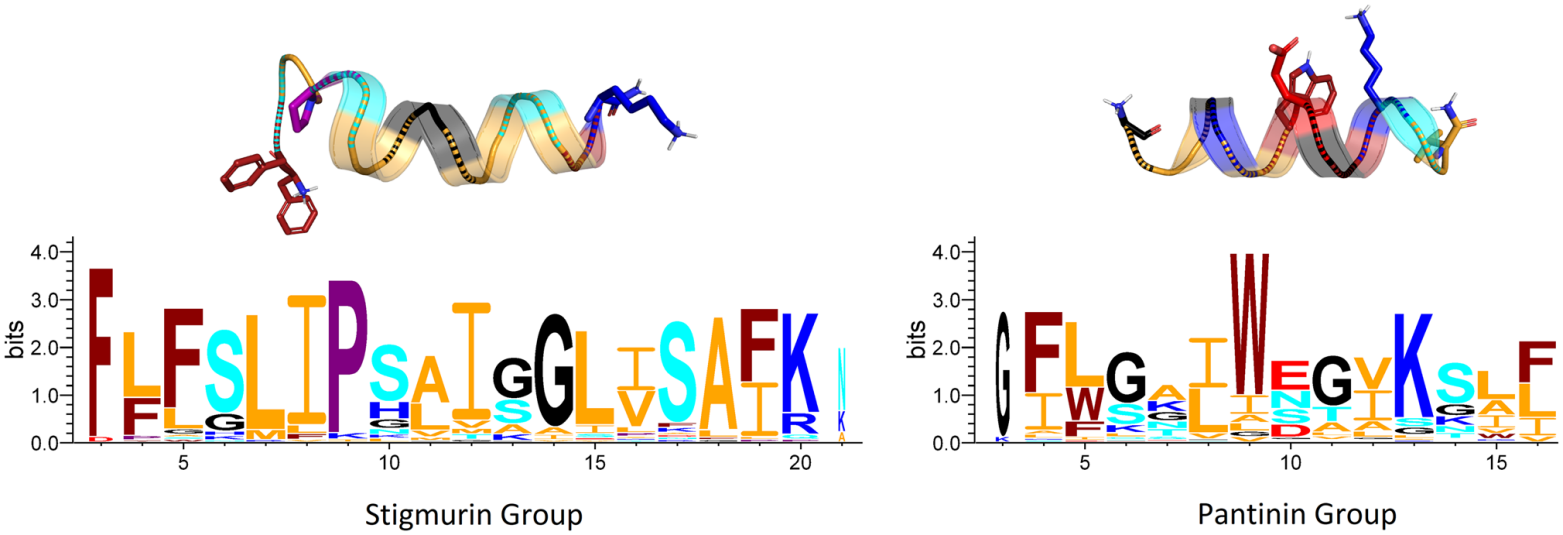
Consensus sequences and typical structures of peptides with similarity to Stigmurin (PDB: 6lv2) and Pantinin-1 (AlphaFold2 model). The logos represent the weighted frequency of each residue in bits per position. The most conserved residues are shown as side chains in each case and include the positive charge at the N-terminus and the amidation at the C-end. Color codes: orange (hydrophobic); cyan (polar); blue (basic); red (acid); maroon (aromatic); purple (proline); black (glycine)

One of the most important characteristics of AMPs is the distribution of hydrophilic and hydrophobic residues along the chain. Since these peptides exert their effects in an aqueous context, the hydrophobic effect facilitates conformational changes that result in the generation of disordered and very dynamic structures. In the VmCT1 peptide and some of its derivatives, the absolute lack of α-helical structures in water has been quantified (Pedron et al. 2019a,b). However, if one considers what we know about one of the best-studied AMPs, i.e., melittin, an effect of pH on the degree of helicity that this peptide assumes in aqueous solution has been verified, as well as the effect of ionic strength in promoting ordered α-helical structures (Ramalingam et al. 1992). Although the detailed mechanism of action of many ssAMPs has not yet been elucidated, it is well established that, as in the case of other widely studied peptides such as melittin, interactions with the membrane surface also facilitate this disorder-to-order transition (*see* Sect. “Surface Charge”). From the perspective of their composition, ssAMPs typically contain around 60% hydrophobic residues (e.g., in the case of the peptide Uy234, a member of the Stigmurin-like group) and up to 80% for the peptide VmCT1, a member of the Pantinin-like group (Fig. 3) (Pedron et al. 2019a, Salazar-Hernandez et al. 2024).

**Fig. 4:**
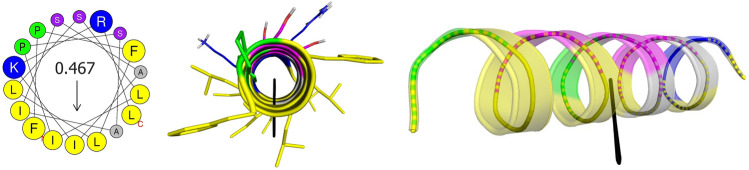
Helical-wheel projections and three-dimensional structure with the calculated HM vector of Uy234 peptide from *Urodacus yaschenkoi*. The hydrophobic moment (μ*H*) was calculated with the Heliquest server (*left*) and it corresponds to a 3D-μ*H* of 4.84 ÅkT/*e* and a θ angle of 95.63° between the vector and the axial axis of the idealized peptide in the 3D-HM server (*right* and *bottom*) (Reiβer et al. 2014)

This amphipathic character, as well as the regularity of the distribution of hydrophobic residues, facilitates the segregation of polar and charged residues on an opposite face in the α-helix. During its adsorption on the membrane, this arrangement of both faces, one hydrophobic oriented to the region rich in the acyl chains of the lipids, and the other hydrophilic in close contact with the aqueous solution and the lipidic polar heads, facilitates a phenomenon of disturbance in lipid packaging (Dathe and Wieprecht 1999). This perturbation will depend both on the relative size of the side chains exposed on each face and on the angle subtended between both faces. Given that many studies indicate that there is a direct relationship between the degree of amphiphilicity and the lytic activity of these peptides, it is necessary to quantify the magnitude of their amphipathic character. For this, two criteria have mainly been used (Fig. 4): (1) the angle subtended by the positively charged helix face (Φ), and (2) the hydrophobic moment (μ*H*).

In a detailed study with the amidated magainin 2 (M2a), Wieprecht et al. (1999) evaluated the effect of charge distribution in terms of this angle but maintaining both the global hydrophobicity of each peptide, as well as its net charge and hydrophobic moments. These authors conclude that in the presence of negative lipids (PG), the lytic capacity of those peptides decreases when the angles approach to 180°. However, in the presence of zwitterionic membranes (PC) and binary mixtures (PC:PG, 3:1), a more homogeneous distribution of both sides (hydrophilic face, Φ= 140–180°) has greater activity compared to peptides where the hydrophilic face decreases to an angle of 80–120°. In an analog study, it was also found that M2a peptides with large Φ angles were more active against ATCC strains of *E. coli*, *P. aeruginosa* and *S. aureus* but decreasing its original specificity (Dathe et al. 1997). In an effort to determine these kinds of subtle effects on the distribution of hydrophilic residues in ssAMPs, an analogous effect was observed in peptides derived from BmKn2 (FIGAIARLLSKIF-NH_**2**_), a member of the Pantinin-like group and identified in the scorpion *Mesobuthus martensii*. In addition to confirming the effect of increasing the cationic charge per se on the antimicrobial activity of this peptide, it was also observed that concentrating the basic residues on the hydrophilic face—reducing the Φ—results in an increase in the hemolytic activity along with a reduction in the MIC against ATCC strains of *E. coli* (Luo et al. 2021a). In any case, as recently proposed (Rawson et al. 2022), the delicate balance that exists between the nonpolar and charged/polarized faces, typical of the structure of ssAMPs, directly affects the bioactivity of these natural agents and must be seriously considered for any rational design of novel improved peptides and future antibiotics.

### Hydrophobic Moment (μ*H*)

The main characteristic of all AMP is its amphiphilicity. This property is directly related to the composition and distribution of each amino acid along the peptide chain. Given that the residues are generally distributed forming two regions or faces along the axial axis of the peptides, we can speak of a hydrophobic face and another with a hydrophilic character. This organization results in a vector effect in an aqueous context or within the lipid bilayer. Thus, the hydrophobic moment (μ*H*) is a reliable measure of the amphiphilic character along a regular structure, such as the α-helix, and represents the segregation degree between nonpolar and polar residues (Eisenberg et al. 1982).

Likewise, the μ*H* measures the probability of finding a peptide in a given position considering the physicochemical context in which it is found. With its correct interpretation, the μ*H* can also help to determine which side of the peptide is best oriented toward the membrane surface and can even explain if it contributes to predicting how each monomer inserts into the bilayer (Table 1) (Reiβer et al. 2014). On the other hand, since it is a vector quantity, it must be defined in terms of its magnitude and its direction. In Newtonian terms, the *momentum*, that is, the product of the mass of the peptide and its velocity with respect to a determined point in the aqueous medium, is defined by the driving force which depends on the repulsion exerted by the hydrophobic side of the peptide with water. In this way, both the length of the vector and the θ angle between the vector and the axial axis of the peptide in its idealized conformation represent the hydrophobicity gradient and can be used to quantify the membranolytic capacity of any AMP. Assuming this principle, the 3D-μ*H* is a geometric parameter that allows estimating the degree of inclination that a given peptide acquires when it is inserted along the membrane normal. AMPs with acute θ angles will be more prone to better insertion into the lipid bilayer, whereas those close to a value of 90° will prefer the interfacial adsorption state on the membrane surface (Strandberg et al. 2020). Thus, those peptides that have a greater propensity to orient themselves transversely tilted along the membrane normal will be more active. In that sense, the case of melittin is paradigmatic, since it has been proven that its partition coefficient in lipid bilayers is high and this correlates quite well with the magnitude of its μ*H*, which is why it has been considered a “surface-seeking helix” (Terwilliger et al. 1982; Thiaudière et al. 1991).

**Table 1:** Short scorpion antimicrobial peptides (ssAMPs) and their main physicochemical properties as a function of the lipid composition in sensitive bacteria

ssAMP	*μ* (Debye)	3D-HM (ÅkT/*e*)	*φ* (kJ/mol)	mBf	Best activity against	Major membrane lipids^a^	MIC (μM)	Hemolysis (%)	References
VmCT1_**NH2**_ (13 aa)	168	7.14	8.34 E3	1.602	*Ps. aeruginosa* *Serratia marcescens* *S. aureus* *Candida tropicalis*	PE, PG, CL (*Ps. a*) PE, PG (*S.m.*) PG, LPG, CL (*S.a.*) PC, PE, PI, PS, PA^b^ (*C.t.*)	0.78 (*Ps.a.*) 1.56 (*S.m.*) 3.12 (*S.a.*) 6.25 (*C.t*)	< 12 (1.56 − 6.25 mM)	Pedron et al. (2017); Bermingham et al. (1970); Kuhn et al. (2015); Deschamps et al. (2021)
Stigmurin_**NH2**_ (17 aa)	110	6.42	1.09 E4	1.691	*S. aureus*, *S. epidermidis* *C. albicans*	PG, LPG, CL (*S.a.*) PC, PE, PI, PS (*C.a.*)	8.68 − 9.38 (*S.a.*) 9.38 (*S.e.*) 34.75 − 37.5 (*C.a.*)	7 (1.1 mM) 9 (17.45 mM) 21 (139.5 mM)	Parente et al. (2018); Kuhn et al. (2015); de Melo et al. (2015); Suchodolski et al. (2020)
TsAP-2_**NH2**_ (17 aa)	110	4.95	9.88 E3	1.654	*S. aureus*	PG, LPG, CL (*S.a.*)	17.30 (*S.a.*)	18 (20 mM)	Guo et al. (2013); Kuhn et al. (2015); de Melo et al. (2015)
Uy234_**NH2**_ (18 aa)	164	4.84	1.15 E4	1.793	*S. aureus*	PG, LPG, CL (*S.a.*)	6.25 (*S.a.*)	7 (140 mM)	Cesa-Luna et al. (2019); Salazar-Hernandez et al. 2024; Kuhn et al. (2015)
Ctriporin_**NH2**_ (19 aa)	136	5.88	1.29 E4	1.772	*A. baumannii* *S. aureus*	PE, CL, PG (*A.b.*) PG, LPG, CL (*S.a.*)	9.9 (*A.b.*) 10 (*S.a.*)	8 (12.5 mM) 27 (25 mM) 45 (50 mM)	Kuhn et al. (2015); Luo et al. (2021a, b); Tao et al. (2021)
Pin2 (24 aa)	345	7.41	1.79 E4	2.127	*Listeria monocytogenes* *E. coli* *M. tuberculosis* *S. aureus*	CL, PG, PI (*L.m.*) PE, PG, CL (*E.c.*) PIM^c^, CL, PE (*M.t.*) PG, LPG, CL (*S.a.*)	18.8 (*L.m.*) 18.8 (*E.c.*) 22 − 33 (*M.t.*) 37.5 (*S.a.*)	18 (3 mM) 83 (12.5 mM) 98 (20 mM)	Rodríguez et al. (2011); Bermingham et al. (1970); Khuller et al. (1982); Mozharov et al. (1985); Belokoneva et al. (2003); Bisbiroulas et al. (2011)
Im5_**NH2**_ (25 aa)	162	8.07	1.72 E4	2.178	*A. baumannii*	PE, CL, PG (*A.b.*)	0.9 (*A.b.*)	18 (6.25 mM) 53 (12.5 mM) 94 (25 mM) 100 (50 mM)	Luo et al. (2021b); Tao et al. (2021)

^a^In order of abundance; ^b^Phosphatidic acid; ^c^Phosphatidylinositol mannosides

Similarly, two peptides derived from Stigmurin, which has antimicrobial activity and is synthesized in the venom gland of the scorpion *Tityus stigmurus*, StigA6 and StigA16, showed an increase in antimicrobial activity against Gram-positive and Gram-negative pathogenic strains, including *Enterobacter cloacae*, *P. aeruginosa*, *S. aureus*, *S. epidermidis*, and yeasts belonging to the genus *Candida*. An in-depth analysis of each peptide indicated that this enhancement in growth inhibitory activity and its anti-proliferative effect is directly related to an increase in the net charge and the μ*H* of the analogous peptides (Parente et al. 2018). Likewise, analogs of Hp1404, discovered in the venom of the scorpion *Heterometrus petersii*, have important antimicrobial activity and show relevant cytotoxicity in multidrug-resistant *P. aeruginosa* strains, which has been directly linked to its high μ*H* value (Kim et al. 2018). The μ*H* for the Uy234 peptide (0.467) (Fig. 4) which is quite similar to the one estimated for melittin from *Apis florea* (0.470) (Eisenberg et al. 1984) has also been related to the efficient antibacterial activity against *S. aureus* (Salazar-Hernandez et al. 2024).

### Net Charge (Z)

Since their discovery, the vast majority of AMPs with antibacterial activity have been described as cationic (Lee et al. 2016; Juhl et al. 2021), even those isolated from scorpion venoms (Harrison et al. 2014; Almaaytah and Albalas 2014; Tarazi 2016). However, few exceptions to this rule have been reported, for example, Peptide T and K12 from *Tityus serrulatus* and *Buthus occitanus*, respectively, which have been described as neutral (Almaaytah et al. 2014), or even some anionic peptides that have also been reported but correspond to sequences too long to be included in the present review (Harris et al. 2009). The net positive charge reported for most of these peptides is based mainly on the presence of lysine and arginine residues, which facilitate their interaction with negatively charged membranes. Many of these biomolecules, particularly those of short-chain, are amidated at the C-terminus, which many authors report as an additional net charge in their studies. Some examples of this are the peptides TsAP-1 and TsAP-2 from *T. serratus* (Guo et al. 2013), VmCT1 from *V. mexicanus* (Pedron et al. 2019a), or Pantinin and their derivatives from *P. imperatus* (Crusca et al. 2018). This way of reporting the net charge of these peptides has generated, however, some confusion.

**Fig. 5:**
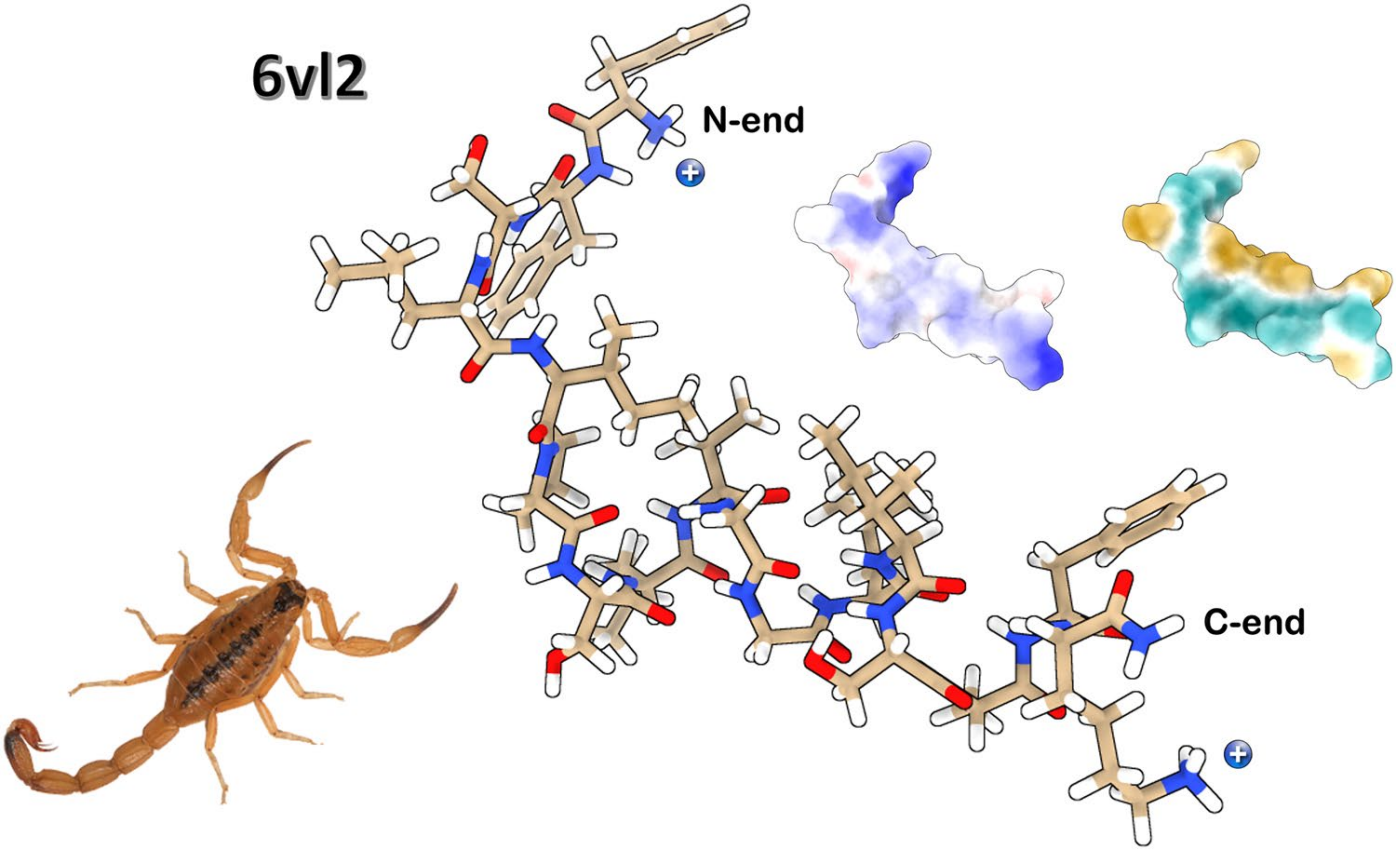
Lower energy structure of Stigmurin (FFSLIPSLVGGLISAFK_NH2__l_ PDB: 6vl2). The side chains of each residue appear in sequence N→C. The net charge (+ 1/ + 1) is determined by the contribution of the N-terminal end at the main chain and another on the side chain of the last residue (K17). Two surface projections are shown exhibiting the electrostatic potential (*left*) and hydrophobicity (*right*). *T. stigmurus* photo credit: iNaturalist Ecuador (https://ecuador.inaturalist.org)

The main contribution to the charge of any oligopeptide or protein is given for the side chains of every residue. Compared to non-amidated forms, peptides amidated at the C-terminus have a higher propensity to form α-helix structures (Mura et al. 2016). In addition, this post-translational modification increases the net charge of the peptide. Figure 5 shows the NMR 3D structure of Stigmurin from the venom of *T. stigmurus*. Stigmurin, which has a net charge of (+ 2) is a clear example of the two types of charges that amidated peptides have, i.e., that present on the lysine or arginine side chains (very rarely histidine) and the ubiquitous positive charge of the unmodified N-terminal end (Fig. 5) (Daniele-Silva et al. 2021).

The effect that a charge on the side chain has, compared to that found on the main chain, is not comparable at all. Sengupta et al. (2005) calculated the free energy of solvation (ΔG_*solv*_) in terms of the electrostatic (ΔG_*elec*_) and nonpolar (ΔG_*np*_) components in synthetic amphipathic peptides. These authors discovered that the energy barrier that a side chain charge must overcome is much lower than the barrier that must be overcome by the charged N- and C-terminal ends to transition from a state adsorbed on the membrane surface to the translocated state across it. This predictive model is perfectly consistent with experimental data for melittin and glycophorin. Likewise, this is verified with what was reported for Spiniferin, a peptide with 13 amino acid residues and amidated at the C-terminal end (ILGEIWKGIKDIL-NH_**2**_). Spiniferin has a net charge of (+ 1) but this charge is located at the N-terminus of residue 1 of the main chain and exhibits very weak antimicrobial activity against both Gram-positive and Gram-negative bacteria, and no activity against human erythrocytes. However, by increasing the net charge by mutating residues E4 and D11 with K4 and N11, Spiniferin-M is obtained, which has a net charge of + 4 (+ 1/ + 3) and this greatly increased its antimicrobial and hemolytic activities (Wu et al. 2014). Something similar was reported for the VmCT1 analogs, where the increase in the net positive charge with basic side chains (Lys and Arg) results in improved peptides in terms of an increase in their antibacterial activities, a better effect against *Trypanosoma* cells, and exhibiting anticancer properties (Pedron et al. 2019b, 2021).

These reports suggest that the presence of positive charges in the form of flexible side chains exposed to the solvent greatly facilitates electrostatic interactions during surface adsorption phenomena and subsequent translocation to the hydrophobic core of the lipid bilayer to facilitate pore formation or an eventual membrane micellization. In energy terms, the contribution of each type of charge (i.e., main chain or side chain) is different (Sengupta et al. 2005). Furthermore, a balance has been noted between hydrophobicity and charge distribution as two factors that act synchronously to enhance the activity of AMPs and reduce the cytotoxic effects in mammalian cells (Yin et al. 2012).

On the other hand, the number of positive charges in side chains is directly related to the antibacterial power in terms of a reduction in the minimum inhibitory concentration (MIC), but this also directly affects the hemolytic activity of AMPs (Jiang et al. 2008). This is clearly noticeable in the case of the peptides StigA6 (net side chain charge =  + 3) and StigA16 (+ 4) derived from Stigmurin (+ 1/ + 1), where an improved antimicrobial effect has been reported against ATCC Gram-negative bacteria (*Escherichia coli*, *E. cloacae*, *P. aeruginosa*), Gram-positive (*S. aureus*, *S. epidermicus*, *E. faecalis*), and yeasts (*Candida albicans*, *C. krusei*, *C. glabrata*) but with 30% of hemolytic activity, whereas Stigmurin exhibits only 3% under the same conditions (Parente et al. 2018). This effect of improving the antimicrobial activity of ssAMPs when the net positive charge is increased by replacing apolar or polar residues with basic residues has also been reported for Hp1404 peptides from the scorpion *Heterometrus petersii* and VmCT1 from *V. mexicanus*. In the first case, a modified ssAMP (Hp1404-T1e, net charge + 1/ + 6) showed great effect against *P. aeruginosa* strains with low toxic and hemolytic activities in mammal cells, compared to the parental peptide (Hp1404; NCZ =  + 1/ + 1) (Kim et al. 2018). In VmCT1 analogs, it is even known that reducing the net cationic charge (NCZ) by including acidic residues such as Glu has an opposite effect in terms of their antimicrobial activity (Pedron et al. 2018). All this evidence indicates that the net charge is one of the most relevant physicochemical parameters in the rational design of AMPs but not the only one to obtain an improved peptide in therapeutic terms.

Regarding the specific position, it is interesting that ssAMPs have their basic side chains preferably toward the C-terminal portion and, at least in the case of the Stigmurin-like peptides, this side chain charge is found in the last residue, typically a lysine (Daniele-Silva et al. 2021). For Pantinin-like peptides (Zeng et al. 2013), the typical basic residue is also a lysine, but this is located at the beginning of the last turn of the α-helix and amidation generally occurs in a hydrophobic residue (Fig. 3). Although ssAMPs have been described with arginine residues in the venom of *Androctonus aeneas* (such as AaeAP1; Du et al. 2015) or even with histidine residues, the latter is linked directly to a highly conserved proline residue in peptides such as AamAP1 and AamAP2 from *A. amoreuxi* (Almaaytah et al. 2012). The preference of lysine over these two residues could respond to an effect of its ionizable methylammonium group, which, unlike the guanidinium group of arginine or imidazolium group of histidine, gives it a slightly less hydrophilic character and consequently could facilitate surface adsorption on membranes. Arginine, on the other hand, being a more hydrophilic residue, would facilitate internalization phenomena that are typical of cell-penetrating peptides (CPPs) (Amand et al. 2012).

Finally, the preference for cationic residues at the C-terminal end of these peptides could not be coincidental; this is because it is proven that such residues also contribute to the stabilization of the helical segments through a partial neutralization effect of the negative dipole in such secondary structures (Armstrong and Baldwin 1993; Resende et al. 2008). Nevertheless, it has also been demonstrated that the pore-forming activity in synthetic peptides increases when they carry a Lys residue at the N-terminus (Strandberg et al. 2020).

### The Electrostatic Potential (φ)

Unlike the effect of net charges, this parameter correlates with the electron density distribution of the peptide in terms of a wavefunction, it brings valuable information about attractive properties and modes of interaction with other molecules for distances of a few Angstroms (Mishra and Kumar 1996). It also depends on the contributions of the distinct chemical groups present in each sequence, particularly those exposed to the solvent. Thus, it is determined the energetics of conformational interactions between the peptide, water, solvated organic molecules, and lipids, where particularly important is the interaction with the polar headgroups containing heteroatoms, such as the –PO_**4**_ group (Scrocco and Tomasi 1973).

Since the main theoretical foundation that determines this parameter depends on Coulomb’s Law, the location of the charges in the peptide is critical because it facilitates, or not, such interactions until reaching an equilibrium in which the charge distribution in a complex system with the lipid membrane becomes stationary. This parameter is also of special interest when considering that the selectivity of AMPs depends mainly on the electrostatic potential established with the surface of biological membranes (Table 1). The electrostatic differential that is established between cationic AMPs and the surface of bacterial membranes is one of the main reasons that explain the high efficiency of these biomolecules as biological control agents (Teixeira et al. 2012). Hence, as bacterial membranes are rich in negatively charged phospholipids (PG, CL, lipid-A), adsorption phenomena on such kinds of surfaces are facilitated (Epand and Epand 2011).

To explain the important electrostatic component of magainin in its binding to biological membranes, Seeling et al. studied these interactions in neutral membranes and concluded that their binding mechanism can be understood through a thermodynamic equilibrium of surface partitioning through the Gouy–Chapman theory where such interaction is driven by the enthalpy of the system (Wieprecht et al. 1999). Under such conditions, the transition toward helical structures from a disordered peptide in the aqueous phase is facilitated. During this transition, on the other hand, the second driving force that facilitates the binding of AMPs to the membrane is the insertion of the nonpolar side chains into the hydrophobic core of the lipid bilayer (Seelig 2004).

**Fig. 6:**
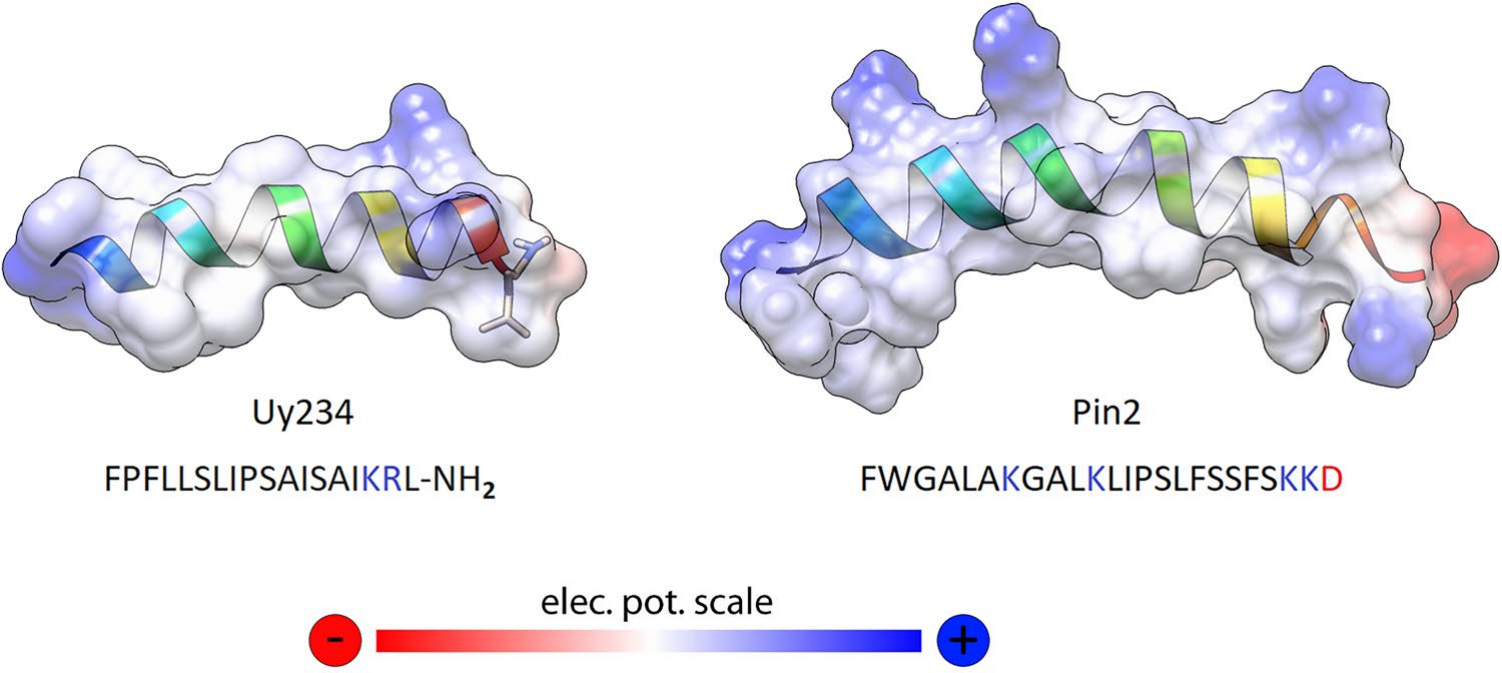
Electrostatic surface potential in ssAMPs. Surface representations with solvent accessibility for the Uy234 peptide (*left*), and pandinin 2, Pin2 (*right*). It is assumed that both relaxed conformations are in an ordered state with the presence of lipids. The color scale covers $\pm$ 5k_B_T/*e*

To verify the basic principles behind this theory, both classical experimental and biocomputing approaches have been followed. In that sense, isothermal titration calorimetry (ITC), zeta-potential measurements, dynamic light scattering (DLC), as well as elaborate molecular dynamics (MD) simulations using the Particle Mesh Ewald (PME) method for electrostatic interactions (Darden et al. 1993) have been particularly useful. In the case of the NK-2, a peptide derived from the cationic region of the NK-lysin, a 78-residue AMP isolated from the pig small intestine, Karmakar et al. (2017) demonstrated a great affinity of NK-2 to DOPG lipid vesicles by detecting a considerable increase in the zeta potential. This confirms the strong electrostatic attraction between cationic NK-2 peptides and negatively charged membranes. It was also found that for this same peptide, the interaction is very weak when interacting with DOPC zwitterionic membranes and DOPC-DOPE binary mixtures. On the other hand, using extensive MD simulations (on the order of μs), pandinin 2 (Pin2) isolated from the venom of *P. imperator* and its analog Pin2GVG exhibit α-helical folding preferentially in dodecylphosphocholine (DPC) and DOPC membranes, respectively (Velasco-Bolom et al. 2020). These results are in quite well agreement with those obtained experimentally and confirm that the electrostatic interactions between peptides and membranes result in a delicate balance between conformational states that are facilitated toward α-helical structures in the presence of specific lipids (Fig. 6). These interactions, in turn, are determined, ultimately and beyond a possible contribution of the curvature of these lipids, by the distribution of the surface charge in both molecular counterparts (Corzo et al. 2001).

### Dipolar Moment (μ)

Given the nature of the covalent bond, where electron pairs are shared forming electron clouds between atomic pairs, and given that each atomic nucleus has a characteristic electronegativity, according to Pauli's original definition, in heteronuclear systems, each nucleus exerts a differential attraction toward the positive charge of the proton cluster inside it (Gupta 2016). Thus, from the valence bond theory, electronegativity is a relative concept and is only defined by the differentials of the product of the magnitude of the charges involved and the distance between the positive and negative centers of a system of charges. However, when trying to determine the effect of electronegativity on organic macromolecules, it is more convenient to associate it with the typical functional groups of organic chemistry and not with the individual atoms (Hol 1985a,b).

In proteins, the peptide bond strongly determines the spatial orientation that the main chain will assume in a physiological context. Given that the peptide bonds assume a planar configuration in *trans*, and that the rotation of the amide plane is practically zero, the two typical configurations of the chains are in the form of α-helical chains or ß-sheets. These configurations are considered ordered because, to be stabilized, the carbonyl and amino functional groups form H-bonds between the oxygen (electronegativity = 3.5) of the carbonyl and the amide (N electronegativity = 3.0) proton of another amino acid residue. In α-helical structures, typical of short-chain AMPs in scorpions, these H-bonds are established every 3.6 amino acid residues per turn. In that configuration, each amide plane is polarized, with the carbonyl vertex being partially negative (δ^**–**^) and the neighboring amino group partially positive (δ^**+**^) (Hol 1985a,b).

Polarity is an electrostatic property quantifying the dipole moment (μ) of a molecule in Debye units (1D = 3.3 × 10^−30^ C m) (Table 1). The polarity of the amide group and its distribution along the peptide are determinants in estimating the degree of polarization of an α-helical structure (Daniele-Silva et al. 2021). Hence, the dipole moment of an α-helical chain can be calculated from the contribution of each peptide unit, *i.e.,* the dipole moments of each amide plane (3.46 D = 0.72 *e*Å). Since in every α-helix the dipole moments of each amide plane are aligned along the axial axis along the helix, it is evident that this parameter has important structural implications and therefore depends on the conformational state of the helix. Likewise, it is known that the axial dipoles of the main chain contribute 97% of the total dipole moment of the helix and that each amide plane can reach 5D if new H-bonds are established at the level of the side chains, for example (Hol et al. 1978).

In terms of peptide functionality, the contribution of the dipole moment has important effects on packing, stabilization of multimeric helical structures through ion–dipole interactions, and shifts in the pKa of specific residues, particularly histidines (Hol 1985a). Given this background, we can assure that one of the main determinants of the activity of α-helical AMPs is their dipole moment, since this is an indicator of the degree of order that the structure acquires and its adsorptive and immersive effect on lipid membranes. From this perspective, it is interesting that the effective dipole moment in lipid monolayers determines the partitioning of melittin at lipid/air interfaces (Wackerbauer et al. 1996). In the case of alamethicin, a 20-residue peptide from the peptaibol family and produced as a secondary metabolite by the ascomycete *Trichoderma viride*, it has an α-helical domain and another one configurated as a 3_**10**_ helix (Fox and Richards 1982) with an effective dipole moment estimated about 67-75D (Schwarz and Savko 1982; Yantorno et al. 1982).

As dipoles tend to arrange in antiparallel, it is interesting that both melittin and synthetic analogs of alamethicin crystallize in antiparallel, suggesting that an adequate dipole moment could be also a requirement for the formation of voltage-dependent multimeric pores (Hanke et al. 1983). Although few studies have focused their efforts on evaluating the contribution that this parameter could have in facilitating the lytic activity of α-helical AMPs, we have reported a significant effect of the dipole moment of the Uy234 peptide from the yellow Brazilian scorpion *U. yaschenkoi* on the stabilization of the secondary structure and its role permeabilizing *S. aureus* membranes (Salazar-Hernandez et al. 2024).

### Helicity

To be active, AMPs must translocate across the membrane in a synergistic effect of adaptation with lipids, thus facilitating the formation of multimeric aqueous pores, destabilizing lipid packaging and promoting micellization phenomena, or acting as transient ionophores and ‘water defects’ (Lee et al. 2016; Juhl et al. 2021; Marquette and Bechinger 2018). As in other AMPs (magainins, melittin, and others) to achieve these effects, a large number of studies indicate that during the first phases of interaction with the membrane, ssAMPs transition from disordered configurations to α-helical structures with different degrees of helicity depending on their amino acid composition because some amino acids are prone to favor helical configurations (for example alanine), while others (in particular proline) destabilize them (Blaber et al. 1993). In addition, it has also been proven that synthetic peptides of the A_**8**_Q_**3**_L_**4**_ family increase their helicity in aqueous medium and during the interfacial partitioning in close relationship with the magnitude of their hydrophobic moments (Fernandez-Vidal et al. 2007).

There is a clear correspondence between the degree of helicity in synthetic AMPs and their antimicrobial/hemolytic activities in model lipid systems. Likewise, the degree of helicity is dependent on length, since long peptides are more likely to form more stable α-helical structures than short ones (Gagnon et al. 2017). It has been demonstrated that longer synthetic peptides are more active in terms of the MIC against bacteria such as *E. coli*, *E. helveticus*, *B. subtilis*, and *S. xylosus* than shorter ones, which also is observed in terms of the hemolytic activities for the same peptides (Strandberg et al. 2020).

**Fig. 7:**
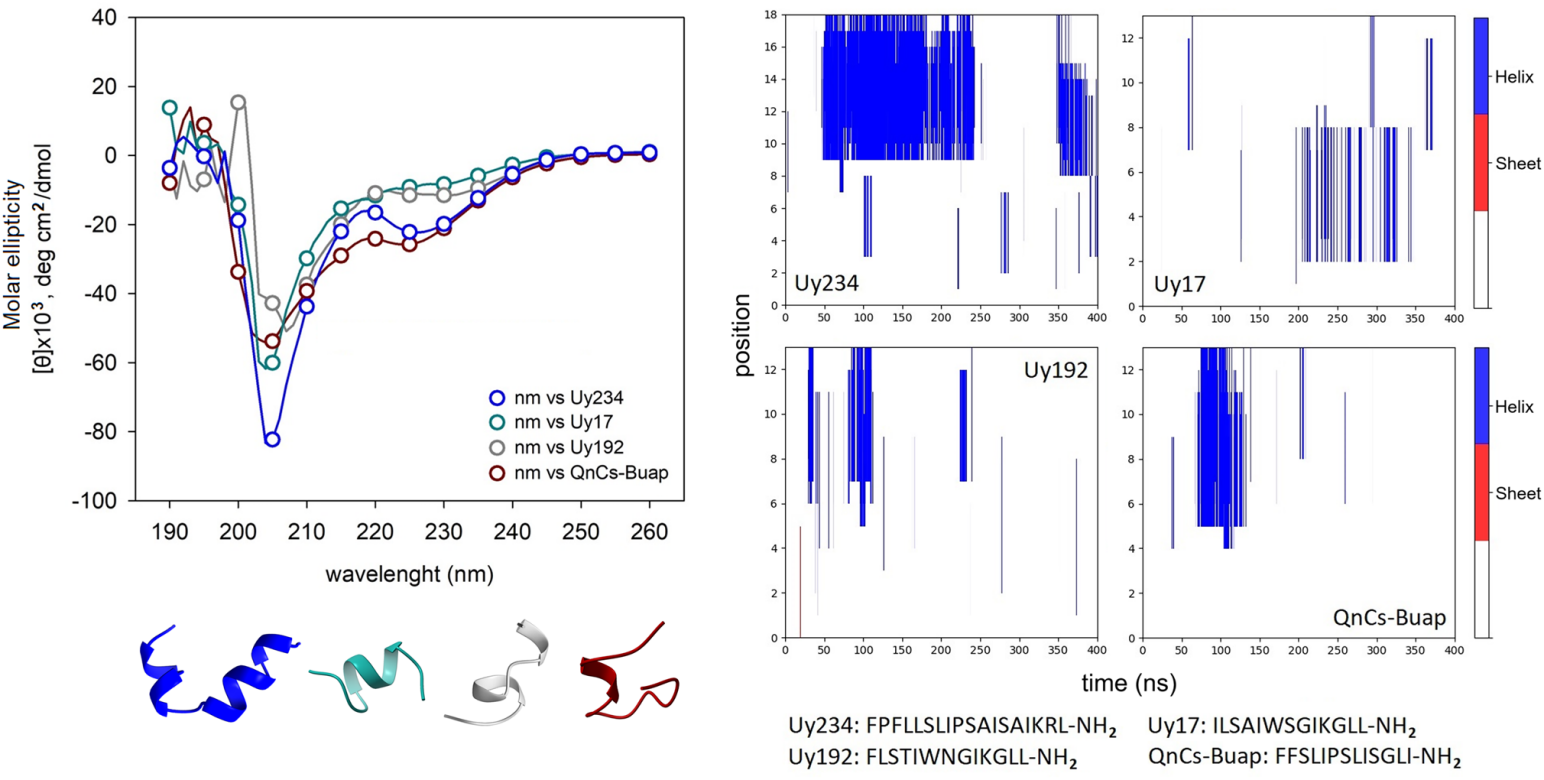
Helicity and conformational dynamics of ssAMPs. CD spectra of three ssAMPs and the hybrid synthetic peptide QnCs-Buap from *U. yaschenkoi* including the propensity to form secondary structures in molecular dynamics simulations with representative structures. CD spectra were obtained according to ref. 11. Both structures and simulations were obtained in the DBAASP database (Pirtskhalava et al. 2021)

These general principles have also been verified in ssAMPs such as those produced by the inland scorpion (*U. yaschenkoi*). Cesa-Luna et al. (2019) have confirmed that the Uy234 (Stigmurin-like group, 18-aa) is significantly more prone to form α-helical structures, while Uy17 and Uy192 (Pantinin-like group, 13-aa), as well as the synthetic peptide QnCs-Buap (FFSLIPSLISGLI-NH_**2**_), which represents a short hybrid between these two families, forms helical structures with difficulty, judging by circular dichroism data (Fig. 7). Again, these structural data correlate quite well with improved activity for Uy234 against several bacterial strains, particularly clinical isolates of *Streptococcus* sp. Uy234, which is more prone to forming stable α-helices, showed greater hemolytic activity, compared to the short peptides and the synthetic hybrid one (Cesa-Luna et al. 2019). Similarly, three isoforms of Pantinin (P1, P2, P3) isolated from *P. imperator* bind preferentially to membranes rich in phosphatidylglycerol (PG) or phosphatidylserine (PS). The partition coefficients in those lipids are directly associated with a greater propensity to be configured as α-helices, particularly in the presence of strongly negative membranes formed with PS, and better in phosphatidylcholine (PC) lipids than in PG lipids. On the other hand, the presence of cholesterol in binary mixtures strongly reduces the transition to α-helical configurations (Crusca et al. 2018). Similarly, another peptide from the Pantinin-like group, IsCT1, isolated from the scorpion *O. madagascariensis* (ILGKIWEGIKSLF-NH_**2**_) is also configured as an α-helix preferentially in the presence of POPC membranes, and less so with POPG (Acevedo et al. 2019). This observation is logical if one takes into consideration that both Pantinin-1 and the IsCT1 peptide have a glutamate residue at the center of the sequence, one turn away from the lysine residue toward the C-terminus (Fig. 3).

VmCT1 (FLGALWNVAKSVF-NH_2_), another ssAMP of the Pantinin-like group, also prefers PC lipids to form α-helices (42%, helical fraction) rather than binary PC:PG mixtures (25%). In this case, the absence of an acidic residue in the analogous position is occupied by a polar residue (Asn) and it is notable that when replacing the Val of the penultimate position with a Tyr residue, the propensity to acquire an α-helical conformation is almost 90% in the same binary model membranes (Pedron et al. 2019a). Again, in this case, the propensity to form α-helical structures correlates quite well with the antimicrobial and anticancer activities of this peptide (Pedron et al. 2018, 2019a,b).

Aminoacidic chirality is also important, albeit the role of lipid chirality remains broadly unknown. In the case of magainin, the substitution of D-enantiomeric residues in specific positions was manifested with a reduction in the permeabilizing activity of the peptide in the presence of liposomes formed with zwitterionic lipids or with a low proportion of negative lipids but not in liposomes rich in PG. The low activity in neutral or slightly negative membranes was correlated with the low helicity of this peptide (Wieprecht et al. 1996). Something similar was reported for the M33-D, a D-enantiomeric version of the synthetic M33 peptide of viral origin, which showed enhanced activity against Gram-negative bacteria such as *S. aureus* and *S. epidermis*. This effect was in turn associated with greater resistance to attack by proteases (Falciani et al. 2012). Although interesting, at the moment none of these observations have been confirmed in ssAMPs and this constitutes a fertile field for future studies. Clearly, more structural studies are needed to confirm the effect of the conformational changes that lead these peptides to acquire ordered configurations. For this purpose, various biophysical techniques with great experimental potential have been used for some time, such as circular dichroism (CD) and its variants, nuclear magnetic resonance (NMR), fluorescence spectroscopy, isothermal titration calorimetry (ITC), and in silico modeling using molecular dynamics (Dathe and Wieprecht 1999; Velasco-Bolom et al. 2020; Aisenbrey et al. 2019).

### Flexibility

Flexibility in proteins and peptides is a topic of increasing relevance to understanding their function, particularly aspects related to the folding of the native structure, the conformational changes associated with its function, and the eventual adaptation to diverse chemical ligands (Halle 2002; Liu and Fang 2013). From a thermodynamic point of view, the effects of flexibility in terms of vibrations, rotations in side chains, folding, allosteric regulation, and ligand binding are those typically associated with the function and dynamism of proteins (Teilum et al. 2009). In the case of short amino acid sequences, typical of AMPs, probably the aspect of greatest interest associated with the effects of flexibility could be the transition from a disordered state in the aqueous phase to an ordered state in close contact with the lipid matrix, as well as the facilitation of their internalization into the membrane to permeabilize or disintegrate it (White and Wimley 1998; Wimley 2010).

As the flexibility parameter is encoded in the amino acid sequence (Schlessinger and Rost 2005; Sonavane et al. 2013), it is possible to classify AMPs as intrinsically rigid or flexible, being long peptides more flexible than short ones in terms of the main B-factor, mBf (Balleza 2023) (Table 1). Using this logic, it has been determined that peptides such as Maculatin 1.1, Brevinin-1, and Pseudin-2 are rigid compared to more flexible ones such as Ranateurin-1, HP(2-20), and CAP18 (Liu and Fang 2013). In that study, it was determined for the first time that rigid peptides have significantly higher Young's moduli than flexible ones, being this module defined in terms of the tensile property associated to the flexural rigidity of the backbone chain. Likewise, the direct relationship that exists between the hydrophilicity and flexibility of numerous AMPs was established, and this in turn with the associated charge density. On the other hand, since it is not so dependent on the composition, the hydrophobic moments do not seem to be directly related to the intrinsic flexibility of the studied AMPs (Liu and Fang 2013). That study and a previous report from the same group also prove that the antimicrobial activity associated with rigid peptides is enhanced by slightly increasing their flexibility, while those AMPs that are too flexible increase their activity by making them slightly more rigid (Liu et al. 2011, Liu and Fang 2013).

The ambiguous effect that flexibility has on the activity of several AMPs has also been reported for specific sequence motifs, where the presence of a central Pro, Gly, or small flexible motifs determines an important structural element in peptides, which can decrease or increase their antimicrobial activity (Juretić et al. 2018; Tuerkova et al. 2020). In Buforin-II and maculatin 1.1, a central Pro residue facilitates their internalization through lipid membranes (Park et al. 2000; Fernandez et al 2013). Furthermore, it has been proven that the synergistic formation of toroidal pores or ‘wormholes’ is facilitated when the peptides are long enough to cross the lipid bilayer and have this Pro-kink motif, but they destabilize the formation of barrel-stave pores. In sum, “peptide flexibility modulates the formation of barrel-stave and toroidal pores” (Tuerkova et al. 2020).

**Fig. 8:**
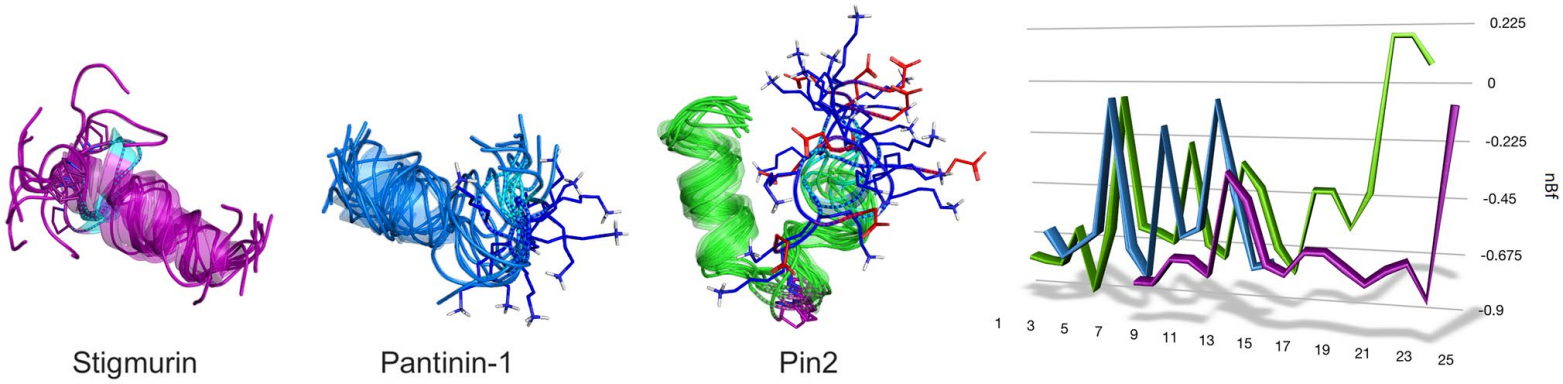
Intrinsic flexibility. Stereoview of a best-fit superimposition of ten models of Stigmurin (17 aa), Pantinin-1 (14 aa) and Pin2 (24 aa) in N-to-C orientation. Pro-6 (*purple*) and Ser-7 (*cyan*) of Stigmurin make the N-terminal end of the peptide more dynamic; on the contrary, the KS motif (positions 11–12, *blue-cyan*) makes the C-terminus of Pantinin-1 more mobile. In Pin2, the presence of Pro-14 (*purple*) and the C-terminal SKKD motif (*cyan-blue-red*) makes the C-terminal region of the peptide more flexible and highly mobile. Flexibility profiles aligned based on the sequence motifs of each peptide are depicted. Estimation of flexibility was reported according to Balleza (2023)

In ssAMPs, the important role of the central Pro in the activity of Pin2 has been proven some time ago. Rodríguez et al. (2011) replaced that residue with hinges of GV, VG, or GVG trying to simulate flexible sequence motifs found in cecropins, oxypinins, and ponicins, concluding that the antimicrobial activity remains as in the parental peptide but the hemolytic activity can be significantly enhanced by substituting the central Pro in particular with the GVG motif. A similar work carried out with Smp24 from Scorpio *maurus palmatus* demonstrated, on the other hand, that the substitution of a central proline for the GVG motif does not alter its hemolytic activity (Harrison et al. 2016). In our previous work with Uy234, we have also found a pivotal role for this central proline, which, due to its high conservation in peptides of the Stigmurin-like group (Fig. 3), as well as in Pin2 analogs, allows us to suggest that this residue determines both the internal bending of the peptide and the facilitation of hydrophobic interactions between the N-terminal end and the acylated chains of anionic lipids (PG) which are more accessible due to the small headgroup of this lipid (Salazar-Hernandez et al. 2024). This proposal could also be supported by a recent study, where it has been possible to simulate Pin2GVG in the presence of zwitterionic lipids (POPC) and it has not been possible to make it more flexible in the way that proline does to enter the hydrophobic core of these membranes (Velasco-Bolom et al. 2020). Likewise, in that study, it was verified—as we also show here—that, unlike Uy234, the region of greatest flexibility of Pin2, as in peptides from the Pantinin-like group, is the C-terminal region (Fig. 8).

**Fig. 9:**
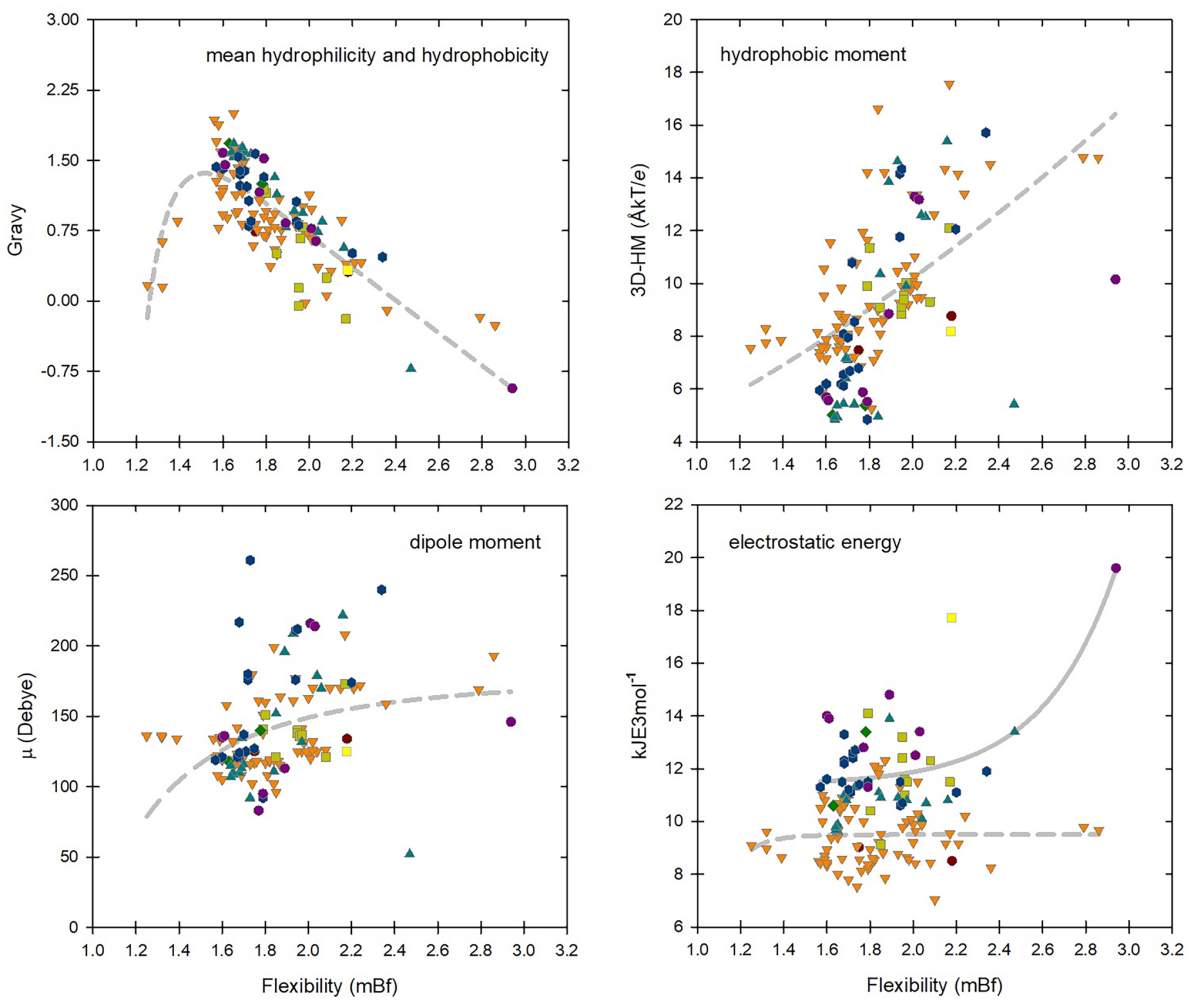
Sequence analysis of 132 scorpion antimicrobial peptides. **A** Intrinsic flexibility (mBf) versus Gravy index (peptide size between 11 and 25 amino acids). **B** Flexibility versus hydrophobic momentum. **C** Flexibility versus dipole moment. **D** Flexibility versus electrostatic energy, where the dashed line represents the best fit for shorter sequences (11–13 aa) and the solid line represents the best fit for longer sequences (14–25 aa). Each data point is defined as a function of peptide size as follows: brown circles (11-aa); orange triangles (13-aa); olive squares (14-aa); green diamonds (16-aa); teal triangles (17-aa); blue hexagons (18-aa); purple circles (19-aa); yellow squares (25-aa)

Finally, since the intrinsic flexibility of an amino acid sequence is a reflection of its chemical composition (Schlessinger and Rost 2005), we took this parameter as a reference to try to establish a correlation between the main physicochemical parameters governing the bioactivity of ssAMPs. In Fig. 9, we show these comparisons and find interesting parallels such as the fact that as ssAMPs acquire flexibility they are generally more hydrophilic and exhibit better hydrophobic moments. Furthermore, although the dipole moment is related to an order parameter in structural terms (see Sect. "Dipolar Moment (μ)") we find that this feature may be dependent on the conformational freedom of each structure. In addition, as the electrostatic potential mainly depends on the presence of charged side chains or polarized, which are associated with high flexibility, some long ssAMPs (14–25 aa) also show high electrostatic energies, whereas this correlation is not as clear in shorter sequences (11–13 aa).

### C-Terminal Amidation

C-terminal amidation is a highly conserved characteristic in peptides with antimicrobial activity present in many metazoan venoms. In scorpions, as in other taxa, enzymatic pathways that promote post-translational modification have been described. The enzymatic modification depends on amidation signal motifs already identified in propeptide sequences (R-X-Gly-K/R or R-X-Gly-K/R-K/R) (Delgado-Prudencio et al. 2019, 2022). The synthesis of amidated peptides has been described in a large number of scorpion venoms mainly through spectroscopic techniques, and when the structure of Stigmurin was reported, the presence of a carboxyamidation of the C-terminal Lys residue was also confirmed (Daniele-Silva et al. 2021). In that report, it is concluded that the modification with this functional group, hand in hand with the presence of basic residues toward the C-terminal end, has an additional effect by neutralizing the negative part of the helical dipole. This facilitates the formation of additional hydrogen bonds throughout the main chain of the oligopeptide. The same has been reported for frog short peptides, phylloseptins (19 aa), where the stabilizing effect of this functional group is maximized by the presence of His residues toward the C-terminus (Resende et al. 2008). It is also well known that amidated peptides, such as frog aurein 1.2 (GLFDIIKKIAESF-NH_**2**_), compared to their carboxylated versions, exhibit enhanced antimicrobial activities and better membrane binding capabilities in terms of the C-terminal hydration state (Shahmiri and Mechler 2020).

Likewise, it was demonstrated that the presence of C-terminal amidation in aurein 2.6 (16 aa) and aurein 3.1 (14 aa) facilitates the formation of α-helical structures and promotes a deeper penetration through the polar heads of PC and PS lipids in close contact with the—PO_**4**_ and—CO_**2**_ groups, respectively (Mura et al. 2016). Thus, the original proposal of a role for the additional charge in amidated peptides seems not to be supported judging by all this experimental and computational evidence. Some authors have proposed that the bioactivity of various AMPs and anticancer peptides in their amidated form would also be related to a reduction in the hydrophilic character of the C-terminal end, which would facilitate its partition in the membrane (Dennison et al. 2009).

**Fig. 10:**
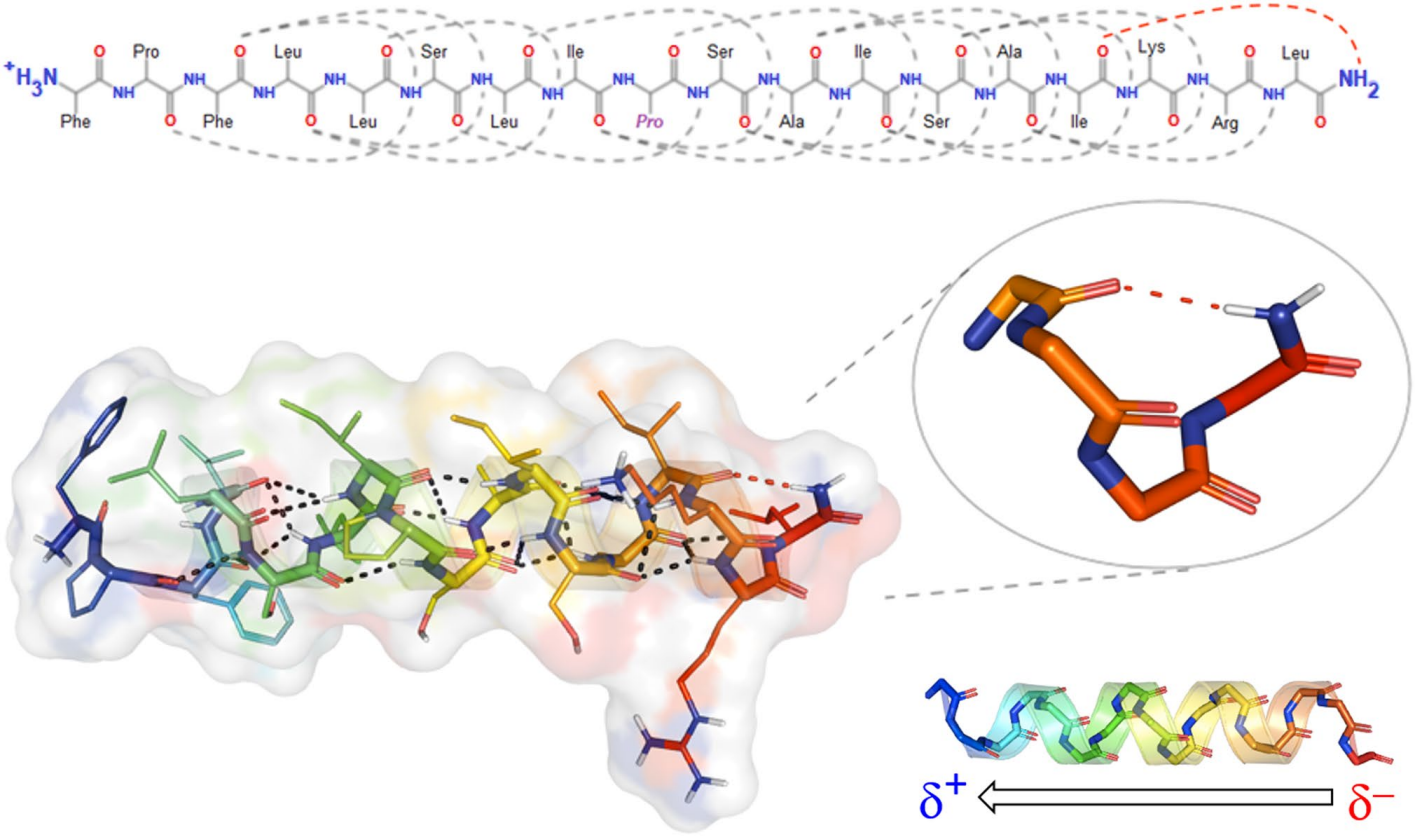
Role of the C-terminal amide group in ssAMPs. Hydrogen bonding network in the backbone of the amidated peptide Uy234 from the scorpion *U. yaschenkoi*. The presence of the − NH_**2**_ group at the C-terminal end, inside the panel, promotes a half turn of Leu18 toward the carbonyl group of Ile15 (Salazar-Hernandez et al. 2024). The alignment of the dipoles in the main chain of an idealized α-helical peptide (Uy234) is included

Although the role of C-amidation in AMPs has been related to greater resistance to degradation by carboxypeptidases, greater structural stability and lower turnover rates (Gutte 1995; Strandberg et al. 2006), information regarding the specific role of this chemical modification in ssAMPs is still scarce. However, it is very notable that most of these short peptides are amidated, and not those that are long such as Pandinin 2 (24 aa) (Fig. 6), Meucin-24 (24 aa) or Meucin-25 (25 aa). Although there are exceptions such as Heterin-1 (43 aa) and Heterin-2 (24 aa) which are also amidated, or Meucin-18 (18 aa) that does not present this modification, the consensus seems to indicate that amidation occurs only in very short peptides (Harrison et al. 2014). This suggests that, given that to stabilize the α-helical structure each residue must establish H-bonds every 3.6 residues per turn, short chains (13–18 aa) such as those that characterize ssAMPs have a higher energetic cost to configure such helices. The presence of the amide group, favoring the formation of additional H-bonds, could reduce the energetic cost of stabilizing α-helices only through the typical H-bonding network of the main chain (Fig. 10). This proposal has been recently verified for the peptide Uy234 and the QnCs-Buap hybrid through ab initio structural modeling and molecular dynamics simulations in the aqueous phase at 300 K and 305 K. Those data confirm the great potential of Uy234 peptide to develop new drugs based on their physicochemical and structural properties (Salazar-Hernandez et al. 2024).

## Physicochemical Parameters Determining the Lipid Bilayer Sensitivity to the Lytic Activity of ssAMPs

Although less studied to date but no less important, this section includes five key parameters that depend on the lipid composition of the biomembranes. Given that the lipid bilayer is the main target where ssAMPs exert their actions, the key parameters to understand these effects include (1) composition, (2) surface charge, (3) lipid packing and fluidity, (4) curvature, and (5) lateral segregation.

### Lipid Composition

Cell membranes are highly diverse in terms of their chemical composition, organization, and electromechanical properties. This often determines cellular function, including the interaction with exogenous agents such as AMPs (Lee et al. 2016; Sani and Separovic 2016; Juhl et al. 2021). As is broadly known, the membranes of eukaryotic cells are significantly different when compared to those of many bacterial or fungal pathogens. To begin with, the level of complexity of a eukaryotic cell in terms of its endomembrane systems is unparalleled in the prokaryotic domain. Furthermore, in terms of chemical composition, eukaryotic cells are enriched in unsaturated lipids with neutral (zwitterionic) headgroups of phosphatidylcholine (PC), a low proportion of anionic lipids (PS, CL, typically in the inner leaflet), and a significant amount of cholesterol and other sterols. In Gram-positive bacteria, the membrane is composed of lipids including significant amounts of anionic lipids in the form of PG, CL, and in many cases lipids with neutral phosphatidylethanolamine (PE) headgroups. In Gram-negative bacteria, the outer membrane contains mainly lipopolysaccharides (LPS) but the inner one is rich in PG, CL, and PE with varying degrees of unsaturation (López-Lara and Geiger 2017). In bacteria, they also include lipids such as phosphatidylinositol (PI), phosphatidic acid (PA), ornithine lipids (OLs), sulfolipids, diacylglyceryl-N,N,Ntrimethylhomoserine (DGTS), glycolipids (GLs), diacylglycerol (DAG), and hopanoids (HOPs) (Sohlenkamp and Geiger 2016). Indeed, given sterols are absent in bacteria, the presence of a great diversity of hopanoids could indicate a highly relevant role for this class of biomolecules (Rohmer et al. 1991).

Mycobacteria membranes are composed of a thick lipophilic layer with mycolic acids (Niederweis et al. 2010). Cell wall includes glycerophospholipids and phosphatidyl-dimannosides, then a layer formed of peptidoglycan-arabinogalactan complex covalently bonded at mycolic acids and long fatty acids (C60–C90) on the outer membrane (Jankute et al. 2015), and interspersed in this layer are lipids such as phthiocerol dimycocerosate (PDIM), trehalose dimycolate (TDM), sulfolipids (SLs), PI mannosides (PIMs), and lipoarabinomannan (LAM) (Adhyapak et al. 2020). In the case of fungal membranes, they are rich in PC, PE, and PI with varying degrees of saturation, in addition to the presence of complex sphingolipids such as inositol-phosphorylceramide (IPCer) and mannosyl-inositolphosphorylceramide (MIPCer). Although cholesterol has been detected in some fungi, the main sterol is ergosterol (Wasser 1977; Santos et al. 2020).

Given this huge compositional diversity in lipids, as well as the current dynamic model to explain the nature of biomembranes, it is not surprising that understanding the actions associated with the bioactivities of ssAMPs depends very closely on the composition of the target membranes. As in most cationic AMPs (Lee et al. 2016), the studies reported at the moment indicate that ssAMPs are also especially sensitive to the presence of anionic lipids PG and less to zwitterionic lipids in the form of PE and PC. Consequently, ssAMPs exhibit their lytic activity or the pore-induction effect mainly when the proportion of PG is high in comparison with those neutral lipids. Such is the case of the smp24, Pantinin1-3, and Pin2 peptides, as well as their cationic derivatives (Nomura et al. 2004; Harrison et al. 2016; Crusca et al. 2018; Velasco-Bolom et al. 2018). Since other relevant biophysical parameters depend ultimately on lipid composition, each of them will be described in the following sections. These parameteres include (1) the effect of surface charge, (2) fluidity and lipid packing, (3) intrinsic curvature, and (4) lateral segregation in lipid domains.

### Surface Charge

The cationic nature of AMPs in the presence of the negative surface of bacterial membranes leads to their localized accumulation. In Gram-negative bacteria, the presence of anionic lipids facilitates adsorption and internalization phenomena through charge exchange mechanisms due to competition with Ca^**2+**^ and Mg^**2+**^ ions that are coordinated with the lipopolysaccharide layer of the outer membrane (Hancock 1984). This second layer constitutes a defensive barrier for antibiotic compounds that cationic peptides are able to penetrate. This was verified with the CEMA peptide, a melittin-cecropin hybrid with two extra positive charges that give it greater permeabilizing activity and makes it more active against Gram-negative bacteria such as *P. aeruginosa* and *E. cloacae* (Piers et al. 1994). However, in Gram-positive bacteria, this accumulation occurs first in the cell wall, rich in teichoic and lipoteichoic acid, covalently linked to N-acetylmuramic acid. The presence of these compounds provides a negatively charged contact surface rich in hydroxyl groups that facilitates the interaction with cationic AMPs. Thus, in Gram-positive bacteria, the presence of a negatively charged mesh with pores of 40 to 80 nm is not an obstacle to the penetration of peptides with dimensions of 50–100 A (Malanovic et al. 2016). Once in contact with the inner membrane, the negative internal transmembrane potential enhances the electrostatic attraction of these cationic AMPs and their subsequent internalization.

**Fig. 11:**
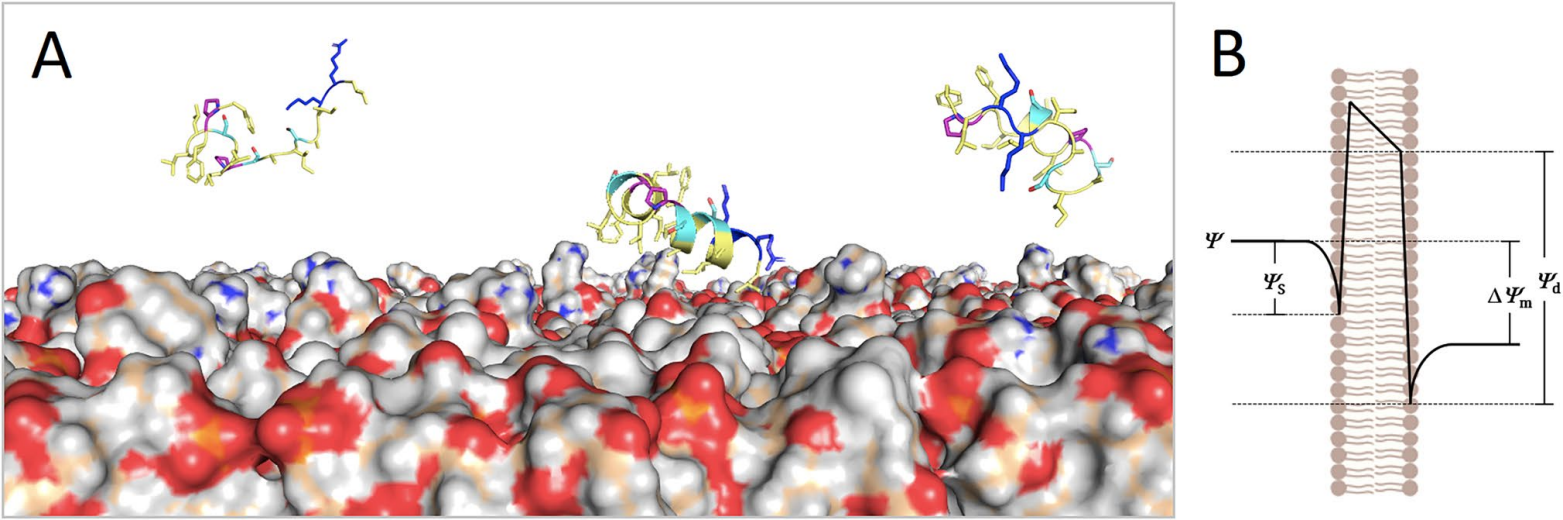
Role of lipid composition in the electrostatic interactions of ssAMPs. **A** Cationic AMPs interact electrostatically and preferentially with negatively charged membranes (rich in PG and CL with minor proportion of PE and PC; oxygens in red). Here, in the aqueous phase, Uy234 are disordered but during adsorption they change to ordered α-helical structures. **B** Electrostatic field distribution across the lipid bilayer, the surface (ψ_**s**_) and transmembrane (Δψ_**m**_) components of dipole potentials (ψ_**d**_) at the interface are depicted. Dotted lines represent the position of each electrokinetic component

Once in contact with the inner membrane, the negative internal transmembrane potential enhances the electrostatic attraction of cationic AMPs and their subsequent internalization (Fig. 11). Thus, it has been shown that the degree of interaction of AMPs with a net cationic charge correlates with the surface negative charge density in lipid membranes (Islam et al. 2023). Furthermore, the free energy profiles associated with these interactions have been calculated and it has been shown that during the conformational transition that leads these peptides to acquire the helical structure, the energy barrier is altered, facilitating the penetration of the helix through the bilayer (Simcock et al. 2021).

Again, the vast majority of the knowledge we have regarding the role of surface charge in the interactions of AMPs with biological membranes comes from what has been discovered in melittin, magainin, cecropin, and aurein (Lee et al. 2016; Islam et al. 2023). In scorpion peptides, the information is still scarce but the general principles seem to be well conserved. For example, UyCT, derived from the venom of the Australian scorpion *U. yaschenkoi*, preferentially interact with binary mixtures that include negative lipids and mimic the membranes of bacterial cells such as *E. coli* (POPE:POPG, 7:3) and *S. aureus* (POPG:TOCL, 3:2). In the presence of such lipids, these peptides have a higher percentage of helicity compared to membranes that simulate the composition of erythrocyte membranes, rich in zwitterionic lipids and cholesterol (POPC:Chol) (Luna-Ramirez et al. 2014). Another study, now with the Ctr or ctriporin, isolated from the venom of *Chaerilus tricostatus* from South Asia, interacts preferentially with anionic micelles of sodium dodecyl sulfate (SDS) forming more flexible helical structures compared to the partially disordered structures that are formed in the presence of zwitterionic *n*-dodecylphosphocholine (DPC) micelles (Bandyopadhyay et al. 2014). This same preference for binding with anionic head groups was also demonstrated in the case of the IsCT1 peptide (Sayyed-Ahmad et al. 2009).

In the case of Pandinin 2 (Pin2) and its analog Pin2GVG, the formation of a large number of H-bonds and electrostatic interactions with polar heads of the anionic lipids POPG has also been confirmed with Molecular Dynamics simulations. However, it is important to highlight that in this case, such a strong electrostatic attraction has a stabilizing effect on the peptide attached to the membrane and opposes its subsequent insertion, since in such simulations, lipid bilayers formed of neutral POPC are preferentially permeabilized (Velasco-Bolom et al. 2018).

Taken together, these results confirm again that these AMPs are disordered in the aqueous phase and are capable of transitioning to helical configurations in the presence of predominantly anionic lipid membranes. This process is facilitated mainly by the electrostatic attraction that is established between the positively charged amino acid residues from those AMPs and the negatively charged phosphate groups of the outer leaflet of the membrane.

### Lipid Packing and Fluidity

The compositional heterogeneity of biological membranes determines many properties related to their function. As a result of the diversity of fatty acids found in bacteria and fungi, it is also possible to find membranes with different degrees of viscoelasticity, which ultimately depend on the presence and proportion of unsaturated fatty acids. These acylated chains have important implications in their degree of packing, since their presence reduces the lipid ordering in membranes (Leekumjorn et al. 2009). The current consensus regarding the mechanism of action of animal cationic AMPs, particularly magainins, is that they are part of a host defense mechanism against several pathogens (McMillan and Coombs 2020). Once embedded in the surface of the membrane, these peptides arranged forming helical structures that cause disturbances in the packaging of lipids. This greatly facilitates the formation of transient defects that can lead to membrane disintegration at high doses (Marquette and Bechinger 2018). Using neutron scattering, it has been shown that aurein 1.2, for example, reduces the lateral diffusion of lipids in the fluid phase, an effect that could induce lipid defects that would facilitate membrane permeabilization (Sharma and Qian 2019). These effects are accompanied by a thinning of the membrane and a modification in the lipid distribution between both leaflets (Rai and Qian 2017). On the other hand, the induction of lipid disorder by the action of several AMPs is well documented by some authors (Killian et al. 1996; Fernandez et al. 2013; Lee et al. 2010, 2014). These effects depend on the accumulation of peptides in the bacterial cell membranes, which interferes with some vital functions, leading to cell death, an effect which is known as the “sand in the gearbox” hypothesis (Roversi et al. 2023).

Another way to evaluate the effects of AMPs on lipid packing is through calorimetric techniques (Prenner et al. 1999). In a study of the three known pantinins (P1, P2, P3), Crusca et al. (2018) managed to obtain experimental evidence of its effect on different model membranes. Thus, it was proven that once in contact with the surface of the lipid bilayer, the peptides come into contact with the hydrocarbon chains of the lipids, thus altering their packing. Likewise, these authors found that those ssAMPs decrease the changes in enthalpy (Δ*H*) and cooperativity of phase transitions in neutral membranes with little affinity for these peptides (DPPC and POPE). In anionic systems, composed of DPPG, an increase in the Δ*H* in the AMP/lipid interaction reveals a disturbance effect on the natural ordering of lipids and a slight decrease in the fluidity on those membranes (Arouri et al. 2013).

### Spontaneous Curvature

Some biophysical parameters of biomembranes directly related with their lipid composition are determined by the packing energy associated to the intrinsic curvature of lipids. The intermolecular packing of lipids is related to the curvature parameter (Brown 2012). The forces of repulsion and attraction inherent in the packing of lipids are energetically balanced in a fluid continuum that can be used during the interaction of AMPs to carry out their lytic activity. This was verified by MD simulations with KIA peptides and model liposomes formed with DMPC and lysoMPC with various degrees of curvature. With this theoretical approach, it was concluded that the spontaneous curvature of the lipid bilayer directly contributes to the formation of aqueous pores where the presence of lipids with high intrinsic positive curvature (lysoMPC) also has a thinning effect on the membrane (Woo and Lee 2017). The molecular shape of an amphiphilic lipid influences its natural propensity for molecular packing and self-assembly. To describe the effective molecular shape, the critical packing parameter (CPP) defines in geometrical terms the relationship between the polar group and the hydrophobic region. This results in cylindrical, conical, or inverted conical lipids which decisively influence the topological parameters of the membranes, relevant in the interaction with AMPs (Paterson et al. 2017).

Experimentally, a curvature-inducing effect has also been confirmed in various AMPs. In Magainin 2, for example, positive curvature is induced in membranes of PG and this facilitates the formation of toroidal pores. The presence of lipids with negative curvature, on the other hand, inhibits pore formation (Matsuzaki et al. 1998). In ssAMPs, the only evidence we have is for Pin2, which also induces curvature tension in lipid bilayers. In the presence of binary mixtures (DOPE:POPC, 6:1), Pin2 reduces the *T*_**H**_ transition temperature from the lamellar liquid-crystalline phase (L_**α**_) to the inverted hexagonal one (H_**II**_) of DOPE, a lipid with negative intrinsic curvature (Nomura et al. 2004). This has been interpreted as an effect of Pin2 to expand the hydrophobic core of the lipid bilayer, which contributes to generating the driving force necessary to deeply insert that peptide into the membrane.

### Lateral Segregation

Lateral lipid segregation is a direct consequence of the lipid composition of membranes and depends directly on the intrinsic curvature of the lipids. In turn, the degree of unsaturation of the acylated chains and the relative size of the polar heads of each lipid determines the curvature parameter. Thus, phospholipids segregate laterally into lipid microdomains or ‘rafts,’ which typically depend on the presence of sterols and that, to achieve energy balance, must establish electrostatic interactions through the membrane normal and adapt to the hydrophobic mismatch. Being key to the function of biological membranes, the lateral segregation of lipids is associated with its dynamics, fluidity, and transport capacities (Lingwood and Simons 2010). Lateral segregation also produces a high degree of disorder in the edges between the lateral lipid domains, which creates packing defects and enhances the susceptibility to AMPs (Paterson et al. 2017).

The effect of AMPs on the lateral secretion of lipids has been studied in aurein 1.2, a short peptide (13 aa) found in the secretions of the frog *Litoria aurea*. Aurein 1.2 induces the lateral segregation of anionic and zwitterionic lipids in an effect similar to the formation of domains below the phase transition for these lipids (Sharma and Qian 2019). This effect of reducing fluidity, promoting the appearance of packing defects, lateral frustrations and topological constraints, enhances the lytic effect of aurein, a common mechanism possibly exhibited by other peptides (Teixeira et al. 2012; Paterson et al. 2017). In ssAMPs, this sensitization effect resulting from the presence of lipids with different transition temperatures has been reported for the peptides Pin1, Pin2, IsCT1, and IsCT2, where the presence of binary mixtures (PC:SM / PC:PE) promotes high permeabilization rates of calcein in fluorescence experiments (Belokoneva et al. 2004).

## QSSAR: The Quest for a Sequence–Structure–Activity Relationship and the Challenge to Engineering or Design Novel Drugs Based on ssAMPs

In a world that is increasingly digitalized and dependent on information technologies, machine learning, and cloud computing, the revolution that artificial intelligence (AI) algorithms have promoted in structural biology is increasingly impacting the rational design of drugs. As explained in the previous sections, ssAMPs and their activities as potential antibiotics are directly associated with a series of parameters that ultimately depend on the structure that these biomolecules acquire in the context of the aqueous/lipid bilayer interface. Given that these peptides carry out conformational transitions during their interaction with membranes, it is evident that optimizing their possible bioactivity will be linked to facilitating their insertion, translocation, and eventual multimerization. It has been suggested that the plasticity of the secondary structure in peptides such as piscidin or His-rich peptides (LAH4) facilitates different types of intermolecular interactions, which explains their mechanisms of action and bioactivities (Campagna et al. 2007; Georgescu et al. 2010). This plasticity in turn depends on factors such as temperature, pH, and solvent characteristics, so a rational design of optimized peptides must consider a variety of environmental parameters and the dynamic configurations that these peptides are capable of perform. Thus, to evaluate experimentally the contribution of some of the physicochemical parameters presented here, the study of the performance of ssAMPs to pH changes, modification of the ionic strength of the aqueous milieu, more detailed molecular dynamics studies or the generation of mutant peptides with specific changes could be decisive in the near future.

The ssAMPs found in scorpion venoms are excellent candidates to plan optimization projects for the development of new antibiotic and antiseptic drugs in general. The idea is to answer the question: is it possible, based on what we know about the effect of the variables discussed here, to establish certain ‘rules’ that these peptides must comply with to be even more effective?

We have known for some time that two of the key factors in the antimicrobial potential of AMPs are the net charge (positive) and amphipathicity (as well as the hydrophobic moment). However, hand in hand with these two parameters, other structural, physicochemical, and even thermodynamic characteristics must contribute more finely to its pharmacological potential. This is also considering the target membrane that is intended to be accessed. Thus, the search for the holy grail in structural biology is the quest for the sequence–structure–activity relationship (QSSAR). Advances in AI-based structure prediction could make it much easier to find this relationship, although one should not blindly trust this technology, which is still in development, leaving aside the need to determine a structure experimentally (Terwilliger et al. 2024). However, the use of these computational algorithms and the increasingly sophisticated visualization and molecular dynamics tools at our disposal are increasingly useful for these purposes.

**Fig. 12:**
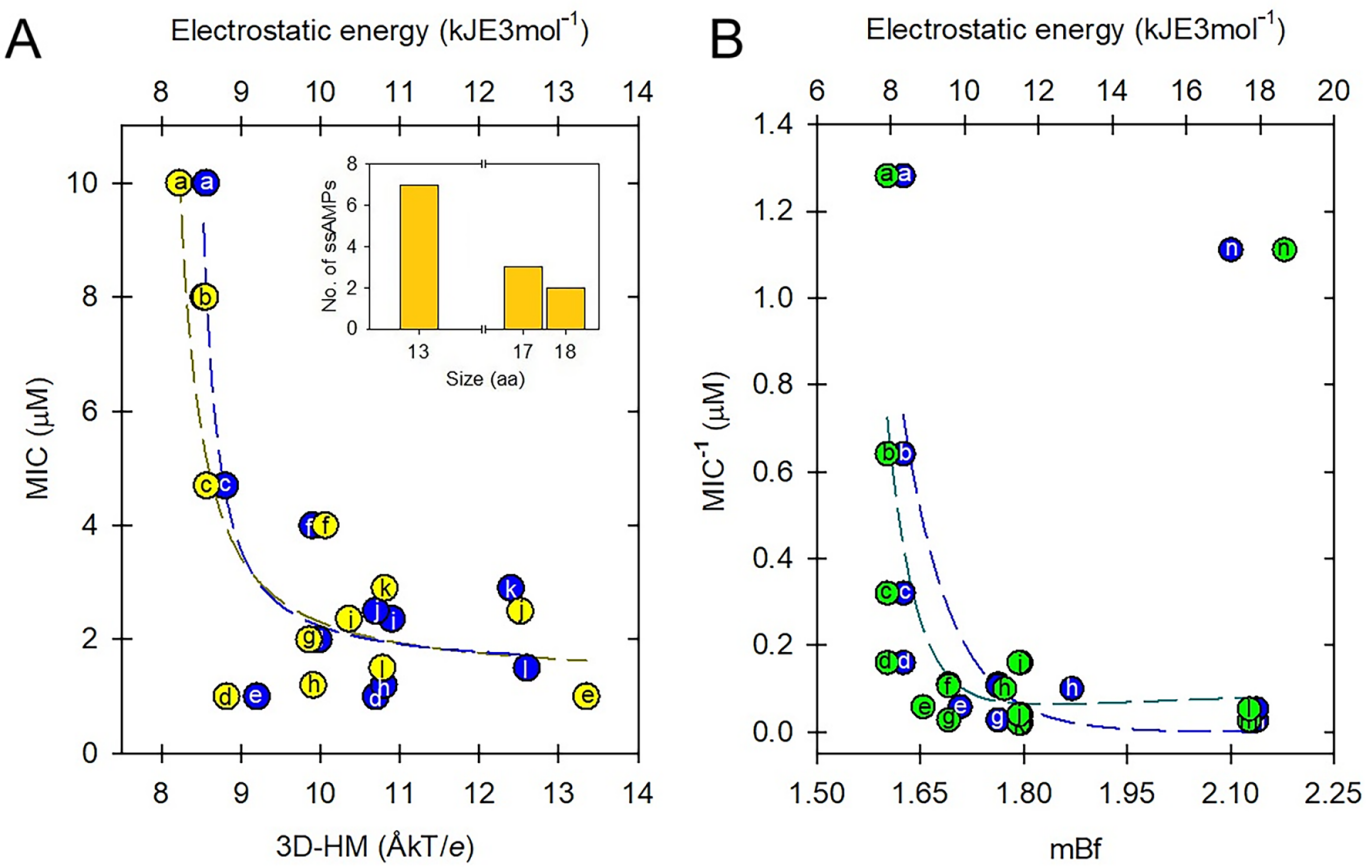
Relationship between the hydrophobic moment (3D-HM), electrostatic energy and intrinsic flexibility (mBf) with the bioactivity of selected ssAMPs.** A** MIC for *S. aureus* versus hydrophobic moment and electrostatic energy of (a) VmCT2, (b) Uy192, (c) VpCT1, (d) UyCT5, (e) IsCT [EK7], (f) IsCT [WL6], (g) IsCT, (h) StigA25, (i) StigA6, (j) TsAP-1, (k) Marcin-18, (l) Megicin-18. The insert indicates the number of peptides according to their size in this distribution. **B** MIC versus intrinsic flexibility and electrostatic energy for (a) VmCT1/*P. aeruginosa*, (b) VmCT1/*S. marcescens*, (c) VmCT1/*S. aureus*, (d) VmCT1/*C. tropicalis*, (e) TsAP2/*S. aureus*, (f) Stigmurin/*S. aureus*, (g) Stigmurin/*C. albicans*, (h) Ctriporin/*S. aureus*, (i) Uy234/*S. aureus*, (j) Uy234/*Bacillus subtilis*, (k) Uy234/*A. baumannii*, (l) Pin2/*Listeria monocytogenes*, (m) Pin2/*S. aureus*, (n) Im5/*A. baumannii*

In addition, the advantage of using predictive algorithms to calculate each of the physicochemical parameters discussed here also contributes to a better design of new peptides with improved activities. Nevertheless, the bioactivities of these peptides cannot be fully understood without considering the contribution that lipid composition of the target cells has on their synergistic effects (Marquette and Bechinger 2018). In terms of sequence, the diversity of ssAMPs is quite wide (Table 3). In this review, we compare 132 peptides and we found two major classes of ssAMPs: those similar to Stigmurins (16–19 aa) and those similar to Pantinins (11–14 aa). A more detailed analysis, selecting some of the best characterized peptides in terms of their biological activities with ESKAPE bacteria such as *S. aureus*, *A. baumannii*, and *P. aeruginosa*, reveals a remarkable relationship between their bioactivity measured as the minimum inhibitory concentration (MIC) and three of the main physicochemical parameters discussed here: (*i*) hydrophobic momentum; (*ii*) electrostatic energy, and (*iii*) intrinsic flexibility. Figure 12A shows that some very small peptides of the Pantinin group (13 aa) are comparatively less effective in terms of the MIC needed to suppress the growth of *S. aureus*. This low efficiency correlates in turn with hydrophobic moments < 10 ÅkT/*e* and electrostatic energies < 10 kJ/mol E3. In contrast, longer peptides, such as some Stigmurin derivatives, Marcin-18 and Megicin-18, are able to inhibit *S. aureus* strains with higher efficiency. This correlates with higher values for these same physicochemical parameters. Likewise, it is well known that increasing the length of peptides generally makes them more flexible (Lee et al. 2004), which we corroborated for the ssAMPs shown in Fig. 12B, where in turn we note that the efficiency of some ssAMPs such as Uy234 or Im5 also correlates with electrostatic energies > 11 kJ/mol E3 and flexibilities that can be high as in the case of the Im5 peptide (25 aa) or low (rigid) as in the case of the VmCT1 peptide (13 aa). These comparisons, however, are not as clear for other peptides, which may suggest that each case should be studied separately, considering the contribution of different variables such as the lipid composition of the target cells, the role of specific residues, or any effect in the peptide oligomerization state during the interaction with the membrane (Chen et al. 2021). Indeed, it has been established that even the most conservative changes in sequence decisively impact the activity of many AMPs, including their selectivity to specific lipids or their propensity to form aggregates (Chen et al. 2021). In any case, it is still not very clear how the most basic physicochemical parameters can determine a priori whether a sequence will be active or not.

Despite their small sizes (13–25 aa), many ssAMPs possess excellent activities against multiple pathogenic bacteria, particularly methicillin-resistant *S. aureus* (MRSA) (Table 2). These activities correlate quite well with some of the main physicochemical parameters characterizing each sequence, such as the hydrophobic moment, net charge, or the White-Wimley index (Table 3). This indicates that such peptides can effectively interact with their membrane targets, despite the known typical thickness of the lipid bilayer (~ 4 nm). Peptide databases such as APD3 or DBAASPR indicate that most non-Cys AMPs found in nature have around 30 residues, which has been proven to be the optimal length capable of forming pores in membranes (Kabelka and Vácha 2015).

**Table 2:** Antimicrobial and hemolytic activities of ssAMPs on *S. aureus*, drug-resistant bacteria and some viruses

ID^a^	Name	Size	STRAIN/virus (MIC)	Hemolysis^b^	References
1465	BmKn2	13-aa	*S. aureus* AB94004 (6.25 µg/mL)	50% at 17.13 µg/mL	Cao et al. (2012a, b)
1466	BmKb1, NDBP4.2, Toxin peptide 6, MeuFSPL1	18-aa	*S. aureus* CGMCC 1.89 (MBC 5.27 µM)	MRBCs (61 ± 2.5% at 3.12 µM)	Gao and Zhu (2018)
2144	Meucin-18, VAP-9	18-aa	*S. aureus* CGMCC 1.89 (MBC 0.87 µM)	MRBCs (100% at 12.5 µM)	Gao and Zhu (2018)
2159	IsCT	13-aa	*S. aureus* KCTC 1621 (2 µM)	41% at 25 µM	Lee et al. (2004)
2160	IsCT [WA6]	13-aa	*S. aureus* KCTC 1621 (> 64 µM)	~ 1% at 25 µM	Lee et al. (2004)
2161	IsCT [WL6]	13-aa	*S. aureus* KCTC 1621 (4 µM)	2% at 25 µM	Lee et al. (2004)
2162	IsCT [EK7]	13-aa	*S. aureus* KCTC 1621 (1 µM)	8% at 25 µM	Lee et al. (2004)
2163	IsCT [WL6, SK11]	13-aa	*S. aureus* KCTC 1621 (2 µM)	2% at 25 µM	Lee et al. (2004)
2164	IsCT [EK7, GP8, SK11]	13-aa	*S. aureus* CCARM 3543 (0.5 µM) MRSA	~ 1% at 25 µM	Lee et al. (2004)
3246	IsCT2f	11-aa	(Not active at 100 µM)	Not active at 100 µM (SRBCs)	Dai et al. (2002)
3247	IsCT2	13-aa	*S. aureus* ATCC 25293 (0.7 µM)	SRBCs (0% at 25 µM)	Dai et al. (2002)
3248	IsCTf	11-aa	(Not active at 100 µM)	Not active at 100 µM (SRBCs)	Dai et al. (2002)
3343	Mastoparan-L	14-aa	*S. aureus* ATCC 292136.3 (12.5 µM)	10% at 100 µM	Moerman et al. (2002)
3353	Hp1090, Um5	13-aa	*S. aureus* DSM 2569 (Not active at 250 µM)	PRBCs (10% at 11.944 µM)	Luna-Ramirez et al. (2017)
3354	Hp1035	13-aa	Hepatitis C virus (HCV) (Not active)	–	Yan et al. (2011)
3475	CT1, VmCT1	13-aa	*S. aureus* ATCC 25923 (10 µM)	12% at 50 µM	Ramírez-Carreto et al. (2012)
3476	CT2, VmCT2	13-aa	*S. aureus* ATCC 25923 (10 µM)	84% at 50 µM	Ramírez-Carreto et al. (2012)
3477	CT1, VsCT1	13-aa	*S. aureus* ATCC 25923 (Not active at 50 µM)	6% at 50 µM	Ramírez-Carreto et al. (2012)
3478	CT2, VsCT2	13-aa	*S. aureus* ATCC 25923 (Not active at 50 µM)	2% 50 µM	Ramírez-Carreto et al. (2012)
3479	CT1-NDBP-5.17, UyCT1	14-aa	*S. aureus* ATCC 25923 (15 µM)	26% at 15 µM	Luna-Ramirez et al. (2013)
3480	CT1-NDBP-5.17, UyCT2	13-aa	*S. aureus* ATCC 25923 (Not active at 100 µM)	7% at 25 µM	Luna-Ramirez et al. (2013)
3481	CT3-NDBP-5.15, UyCT3, OcyC1, NDBP-5.7	13-aa	*S. aureus* ATCC 25923 (10 µM)	20% at 10 µM	Luna-Ramirez et al. (2013)
3482	CT5-NDBP-5.16, UyCT5	13-aa	*S. aureus* ATCC 25923 (1 µM)	0% at 2 µM	Luna-Ramirez et al. (2013)
3489	Pantinin-2	13-aa	*S. aureus* AB94004 (48 µM)	8% at 16 µM	Zeng et al. (2013)
3490	Pantinin-1	14-aa	*S. aureus* AB94004 (8 µM)	21% at 64 µM	Zeng et al. (2013)
3491	Pantinin-3	13-aa	*S. aureus* AB94004 (16 µM)	70% at 16 µM	Zeng et al. (2013)
3599	CT1	14-aa	*S. aureus* AB 94004 (12.5 µg/mL)	ND	Yuan et al. (2010)
3615	CT2, Um3	14-aa	*S. aureus* AB94004 (6.25 µg/mL)	50% at 80.3 µg/mL	Cao et al. (2012a, b)
3752	Mucroporin	17-aa	*S. aureus* AB 94004 (25 µg/mL)	ND	Dai et al. (2008)
3753	Mucroporin-M1, Mucroporin [G3,11R, P6K, G10K]	17-aa	*S. aureus* AB94004 (5 µg/mL)	–	Dai et al. (2008)
3754	Imcroporin	17-aa	*S. aureus* AB94004 (20 µg/mL)	–	Zhao et al. (2009)
3884	AamAP1	18-aa	*S. aureus* NCTC 10788 (20 µM)	100% at 120 µM	Almaaytah et al. (2012)
3885	AamAP2	18-aa	*S. aureus* NCTC 10788 (20 µM)	100% at 100 µM	Almaaytah et al. (2012)
3935	Ctriporin	19-aa	*S. aureus* AB94004 (5 µg/mL)	10% at 30 µg/mL	Luo et al. (2021a, b); Fan et al. (2011)
4297	TsAP-1	17-aa	*S. aureus* NCTC 10788 (120 µM)	6.48% at 160 µM	Guo et al. (2013)
4298	TsAP2	17-aa	*S. aureus* NCTC 10788 (5 µM)	100% at 80 µM	Guo et al. (2013)
4299	TsAP-1 [S7K] [G10K] [G11K] [S12I] [S14K]	17-aa	*S. aureus* NCTC 10788 (2.5 µM)	EqRBCs (28% at 5 µM)	Guo et al. (2013)
4300	TsAP-2 [G7K] [G10K] [G11K] [S14K]	17-aa	*S. aureus* NCTC 10788 (5 µM)	EqRBCs (28% at 5 µM)	Guo et al. (2013)
4330	Hp1036	13-aa	HSV-1 (IC_50_ 0.43 ± 0.09 µM) Inhibition of the initial infection	50% at 34.91 ± 0.47 µM	Hong et al. (2014)
4331	Hp1239	13-aa	HSV-1 (IC_50_ 0.41 ± 0.06 µM) Inhibition of the initial infection	50% at 33.32 ± 0.96 µM	Hong et al. (2014)
4572	Kn2-7	13-aa	*S. aureus* AB94004 (3.13 µg/mL)	50% 90.27 µg/mL	Cao et al. (2012a, b)
7227	AamAP1 [S4K, H8K, G11, 12 K, A16K]	18-aa	*S. aureus* ATCC 29213 (5 µM)	0% at 5 µM	Almaaytah et al. (2014)
8151	AaeAP1	19-aa	*S. aureus* NTCC 10788 (4 µM)	100% at 32 µM	Du et al. (2015)
8152	AaeAP2	19-aa	*S. aureus* NCTC 10788 (16 µg/mL)	EqRBCs (100% at 64 µg/mL)	Du et al. (2015)
8153	AaeAP1 [S4,8,15 K; A11K, N19K]	19-aa	*S. aureus* NCTC 10788 (4 µg/mL)	EqRBCs (100% at 32 µg/mL)	Du et al. (2015)
8154	AaeAP2 [S4,8,15 K; A11K, N19K]	19-aa	*S. aureus* NCTC 10788 (4 µg/mL)	EqRBCs (100% 64 µg/mL)	Du et al. (2015)
8199	Stigmurin	17-aa	*S. aureus* ATCC 29213 (8.68 µM)	20% at 139.5 µM	de Melo et al. (2015)
8437	VpAmp1.0	19-aa	*S. aureus* ATCC 25923 (2.5 µM)	50% at 9.2 µM	Ramirez-Carreto et al. (2015)
8438	VpAmp1.1	17-aa	*S. aureus* ATCC 25923 (5 µM)	50% 33.7 ± 2.4 µM	Ramirez-Carreto et al. (2015)
8864	Hp1404	14-aa	*S. aureus* ATCC 25923 (6.25 µg/mL)	10% at 100 µg/mL	Li et al. (2014)
9867	ToAP3, Im-4	17-aa	*S. aureus* NBRC 13276 (5–10 µM)	SRBCs (50% at > 30 µM)	Miyashita et al. (2017)
9868	NDBP-4.23, TsAP-2, TcAP-4, Tbah00286	17-aa	*C. albicans* SC5314 (50 µM)	48% 100 µM	Guilhelmelli et al. (2016)
9869	ToAP1	17-aa	*C. albicans* SC5314 (50 µM)	48% at 100 µM	Guilhelmelli et al. (2016)
9870	OcyC2, NDBP-5.8	14-aa	*C. albicans* SC5314 (100 µM)	–	Guilhelmelli et al. (2016)
9945	Uy17	13-aa	*S. aureus* ATCC 25923 (MBC 23.2 µM)	< 5% at 380 µM	Cesa-Luna et al. (2019)
9946	Uy192	13-aa	*S. aureus* ATCC 25923 (MBC 42.4 µM)	< 5% at 380 µM	Cesa-Luna et al. (2019)
9947	Uy234	18-aa	*S. aureus* ATCC 25923 (MBC 29.6 ± 25 µM)	25% at 370 µM	Cesa-Luna et al. (2019)
9951	Um2	19-aa	*S. aureus* DSM 2569 (Not active at 250 µM)	PRBCs (10% at 2.36 µM)	Luna-Ramirez et al. (2017)
9952	Um4	13-aa	*S. aureus* DSM2569 (15 µM)	9% at 100 µM	Luna-Ramirez et al. (2017)
9953	UyCT3 [L2F, S3G], D1	13-aa	*S. aureus* ATCC 25923 (MBC 29.6 ± 25 µM)	25% at 370 µM	Luna-Ramirez et al. (2017)
9954	Uy192 [G11S, L13F], D2	13-aa	*S. aureus* DSM 2569 (8 µM)	PRBCs (10% at 2.94 µM)	Luna-Ramirez et al. (2017)
9955	UyCT1 [E8K, G9P, N12K], D4	14-aa	*S. aureus* DSM 2569 (Not active at 250 µM)	PRBCs (Not active at 100 µM)	Luna-Ramirez et al. (2017)
9956	UyCT1 [W7L, N12K], D5	14-aa	*S. aureus* DSM 2569 (Not active at 250 µM)	PRBCs (10% at 110.86 µM)	Luna-Ramirez et al. (2017)
9957	UyCT1—3 K, D11	17-aa	*S. aureus* DSM 2569 (Not active at 250 µM)	PRBCs (10% at 39.76 µM)	Luna-Ramirez et al. (2017)
10158	NDBP-5.5	13-aa	*Mycobacterium abscessus* ssp. *massiliense* (MBC 200 µM)	10% at 611.8 µM	Trentini et al. (2017)
10462	IsCT1 [I5,9A]	13-aa	*S. aureus* ATCC 29213 (> 100 µg/mL)	10% at 100 µg/mL	de la Salud et al. (2017)
10463	IsCT1 [I5,9 V]	13-aa	*S. aureus* ATCC 29213 (> 100 µg/mL)	8% at 100 µg/mL	de la Salud et al. (2017)
10464	IsCT1 [I5,9L]	13-aa	*S. aureus* ATCC 29213 (50 µg/mL)	80% at 50 µg/mL	de la Salud et al. (2017)
10465	IsCT1 [K10E]	13-aa	*S. aureus* ATCC 29213 (> 100 µg/mL)	0% at 100 µg/mL	de la Salud et al. (2017)
10466	IsCT2 [F2L; I5,9A]	13-aa	*S. aureus* ATCC 29213 (> 100 µg/mL)	5% at 100 µg/mL	de la Salud et al. (2017)
10467	IsCT2 [F2L; I5,9 V]	13-aa	*S. aureus* ATCC 29213 (> 100 µg/mL)	18% at 100 µg/mL	de la Salud et al. (2017)
11211	MeuFSPL-2	18-aa	*S. aureus* CGMCC 1.89 (LC 3.14 µM)	MRBCs (100% at 12.5 µM)	Gao and Zhu (2018)
11223	Spiniferin	13-aa	*S. aureus* AB 94004 (> 82 µM)	Not active at 48 µM	Wu et al. (2014)
11224	Spiniferin [E4K, D11N]	13-aa	*S. aureus* AB94004 (12 µM)	1.7% at 6 µM	Wu et al. (2014)
11270	Stigmurin [S7K, G10K], StigA6	17-aa	*S. aureus* ATCC 29213 (2.34 µM)	40% at 75 µM	Parente et al. (2018)
11271	Stigmurin [S3,7 K; G10K], StigA16	17-aa	*S. aureus* ATCC 29213 (2.34 µM)	30% at 75 µM	Parente et al. (2018)
12239	HAP-1 (1–19)	19-aa	*S. aureus* AB94004 (not active at 80 µM)	not active at 80 µM	Shi et al. (2018)
12417	Stigmurin [G10,11 K; S14K], StigA25	17-aa	*S. aureus* ATCC 29213 (1.2 µM)	18.5% at 9.4 µM	Amorim-Carmo et al. (2019)
12418	Stigmurin [S3,6,14 K; G10,11 K], StigA31	17-aa	*S. aureus* ATCC 29213 (2.3 µM)	11.2% at 9.4 µM	Amorim-Carmo et al. (2019)
12573	AamAP1[P7R]; A3 [H8K]	18-aa	*S. aureus* ATCC 29213 (5 µM)	5.1% at 10 µM	Almaayah et al. (2018)
13647	AcrAP1, AP1-Z1	18-aa	*S. aureus* NCTC 10788 (8 µM)	EqRBCs (100% at 64 µM)	Du et al. (2014)
13648	AcrAP1 [S4K, H8K, S11K, S15K]	18-aa	*S. aureus* NCTC 10788 (4 µM) (MBC 32 µM)	EqRBCs (100% at 32 µM)	Lee et al. (2018)
13649	AcrAP2	18-aa	*S. aureus* NCTC 10788 (8 µM) (MBC 32 µM)	EqRBCs (100% at 64 µM)	Du et al. (2014)
13650	AcrAP2 [S4K, N8K, S11K, S15K]	18-aa	*S. aureus* NCTC 10788 (4 µM) (MBC 32 µM)	EqRBCs (100% at 32 µM)	Du et al. (2014)
14497	QnCs-Buap	13-aa	*S. aureus* ATCC 25923 (MBC > 353.1 µM)	< 5% at 380 µM	Luna-Ramirez et al. (2013)
14624	Marcin-18	18-aa	*S. aureus* P1389 (2.9 µM)	–	Liu et al. (2018)
14625	Megicin-18	18-aa	*S. aureus* P1389 (1.5 µM)14,625	–	Liu et al. (2018)
14626	VmCT1 [G3R]	13-aa	*S. aureus* ATCC 29213 (1.6 µM)	0% at 1.6 µM	Pedron et al. (2019a)
14627	VmCT1 [N7R]	13-aa	*S. aureus* ATCC 29213 (1.6 µM)	0% at 3.1 µM	Pedron et al. (2019a)
14634	VmCT1 [S11R]	13-aa	*S. aureus* ATCC 29213 (3.1 µM)	0% at 3.1 µM	Pedron et al. (2019a)
14635	VmCT1 [F1G]	13-aa	*E. coli* SBS 363 (50 µM)	0% at 50 µM	Pedron et al. (2019a)
14636	VmCT1 [V8P]	13-aa	*E. coli* SBS 363 (50 µM)	0% at 50 µM	Pedron et al. (2019a)
14637	VmCT1 [A9L]	13-aa	*S. aureus* ATCC 29213 (0.8 µM)	0% at 1.6 µM	Pedron et al. (2019a)
14638	VmCT1 [A9F]	13-aa	*S. aureus* ATCC 29213 (0.8 µM)	0% at 0.8 µM	Pedron et al. (2019a)
14639	VmCT1 [V12L]	13-aa	*S. aureus* ATCC 29213 (0.8 µM)	0% at 3.1 µM	Pedron et al. (2019a)
14640	VmCT1 [V12Y]	13-aa	*S. aureus* ATCC 29213 (3.1 µM)	0% at 6.3 µM	Pedron et al. (2019a)
15179	Mucroporin (7–17)	11-aa	No virucidal Activity	–	Li et al. (2011)
15558	Eval418	13-aa	HSV-1 (IC_50_ 2.48 µg/mL)	VCs (50% toxicity at 68.50 µg/mL)	Zeng et al. (2018)
15559	Eval418-FH2	13-aa	HSV-1 (IC_50_ 1.50 µg/mL)	VCs (50% toxicity at 27.60 µg/mL)	Zeng et al. (2018)
15560	Eval418-FH3	13-aa	HSV-1 ((IC_50_ 1.01 µg/mL)	VCs (50% toxicity at 26.83 µg/mL)	Zeng et al. (2018)
15561	Eval418-FH4	13-aa	HSV-1 (IC_50_ 0.87 µg/mL)	VCs (50% toxicity at 27.58 µg/mL)	Zeng et al. (2018)
15562	Eval418-FH5	13-aa	HSV-1 (IC_50_ 0.86 µg/mL)	VCs (50% toxicity at 106.7 µg/mL)	Zeng et al. (2018)
16149	MK049518	18-aa	*S. aureus* AB94004 (12.5 µg/mL)	–	Li et al. (2020)
16150	MK049518 [S7,11,15 K]	18-aa	*S. aureus* AB94004 (3.13 µg/mL)	HHCs (90% cell death 12.5 µg/mL)	Li et al. (2020)
16151	MK049518 [G3,6,10 K; S7,11,15 K]	18-aa	*S. aureus* AB94004 (3.13 µg/mL)	HHCs (25% cell death 12.5 µg/mL)	Li et al. (2020)
16788	VmCT1 [F1K]	13-aa	*S. aureus* ATCC 29213 (50 µM)	< 5% at 12.5 µM	Pedron et al. (2019b)
16789	VmCT1 [A9K]	13-aa	*S. aureus* ATCC 29213 (> 50 µM)	< 5% at > 100 µM	Pedron et al. (2019b)
16790	VmCT1 [F1K, V12K]	13-aa	*S. aureus* ATCC 29213 (> 50 µM)	< 5% at > 100 µM	Pedron et al. (2019b)
16791	VmCT1 [G3K, N7K]	13-aa	*S. aureus* ATCC 29213 (0.8 µM)	< 5% at 1.6 µM	Pedron et al. (2019b)
1692	VmCT1 [G3K, S11K]	13-aa	*S. aureus* ATCC 29213 (0.8 µM)	< 5% at 3.1 µM	Pedron et al. (2019b)
16793	VmCT1 [N7K, S11K]	13-aa	*S. aureus* ATCC 29213 (0.8 µM)	< 5% at 3.1 µM	Pedron et al. (2019b)
16794	VmCT1 [G3K, N7K, S11K]	13-aa	*S. aureus* ATCC 29213 (0.8 µM)	< 5% at 3.1 µM	Pedron et al. (2019b)
1726	Im5	25-aa	*A. baumannii* (0.9 µM)	17% at 12.5 µM	Luo et al. (2021b)
17527	Im6	16-aa	*S. aureus* (5–10 µM)	84% at > 30 µM	Miyashita et al. (2017)
18163	VpCT1	13-aa	*S. aureus* ATCC 25923 (4.7 µM)	50% at 10.5 ± 0.4 µM	Jimenez-Vargas et al. (2021)
18164	VpCT2	13-aa	*S. aureus* ATCC 25923 (12.5 µM)	50% at 10.8 ± 0.4 µM	Jimenez-Vargas et al. (2021)
18165	VpCT3	13-aa	*S. aureus* ATCC 25923 (> 100 µM)	50% at 83.7 ± 1.8 µM	Jimenez-Vargas et al. (2021)
18166	VpCT3 [I6W]	13-aa	*S. aureus* ATCC 25923 (> 100 µM)	50% at 37.9 ± 0.4 µM	Jimenez-Vargas et al. (2021)
18167	VpCT4	16-aa	*S. aureus* ATCC 25923 (9.3 µM)	50% at 4.8 ± 0.7 µM	Jimenez-Vargas et al. (2021)
18168	VpCTconsensus	13-aa	*S. aureus* ATCC 25923 (37.5 µM)	50% at 39.9 ± 0.4 µM	Jimenez-Vargas et al. (2021)
18292	Ctri9594	14-aa	*S. aureus* AB94004 (25 µg/mL)	–	He et al. (2021)
18735	Checacin1 (1–11)	11-aa	*S. aureus* ATCC 33592 (> 50 µM)	CKCs (not active at 100 µM)	Krämer et al. (2022)
18736	Checacin1 (12–25)	14-aa	*S. aureus* ATCC 33592 (> 50 µM)	CKCs (not active at 100 µM)	Krämer et al. (2022)
20289	IsCT [E7P]	13-aa	*S. aureus* ATCC 12600 (64 µM)	< 5% at 128 µM	Oliveira et al. (2021)
20290	IsCT [G3K, G8P]	13-aa	*S. aureus* ATCC 12600 (> 128 µM)	< 5% at 128 µM	Oliveira et al. (2021)
20291	IsCT [I1A; G3,8 K; I5F]	13-aa	*S. aureus* ATCC 12600 (8 µg/mL)	< 5% at 32 µM	Oliveira et al. (2021)
20292	IsCT [I1A, I5F, E7P, G8K]	13-aa	*S. aureus* ATCC 12600 (> 128 µM)	< 5% 128 µM	Oliveira et al. (2021)
20293	IsCT [G3K,E7K,I9K]	13-aa	*S. aureus* ATCC 12600 (> 128 µM)	< 5% at 128 µM	Oliveira et al. (2021)
20294	IsCT [G3K, E7K, G8P, I9K]	13-aa	*S. aureus* ATCC 12600 (> 128 µM)	< 5% at 128 µM	Oliveira et al. (2021)
21142	Hp1470	13-aa	*S. aureus* AB94004 (6.25 µg/mL)	–	Li et al. (2023)
21411	TtAP-2	17-aa	*S. aureus* ATCC 43300 (25 µg/mL)	50% at 31 µg/mL	Mechkarska et al. (2023)

*MRBCs* Mice red blood cells; *ND* Not determined; *NDBP* Non-disulfide-bridged peptide; *PRBCs* Pig red blood cells; *SRBCs* Sheep erythrocytes; *VCs* Vero cells

^a^Database of Antimicrobial Activity and Structure of Peptides (DBAASPR)

^b^Data obtained in human red blood cells or: CKC: Madin-Darby canine kidney cells; EqRBCs: Horse erythrocytes; HHCs: Human hepatocyte cells; MBC: Minimal Bactericidal Concentration

**Table 3:** Physicochemical properties composition of short scorpion antimicrobial peptides (ssAMPs)

Accession^a^	Name	Sequence	GRAVY	NZC	µH	3D-HM (ÅkT/*e*)	θ(°)	μ(D)	Total electrostatic energy (kJ/mol)	mBf	W-W INDEX (kcal/mol)	BOMAN INDEX (kcal/mol)	Therapeutic Index^c^
1465 (Q6JQN2)	BmKn2	FIGAIARLLSKIF-NH_2_	1.59	(+ 1/ + 2)	0.76	10.54	125.37	135	1.08 E4	1.59	–2.03	–0.86	5.46
1466 (Q718F4)	BmKb1, NDBP-4.2, Toxin peptide 6, MeuFSPL-1	FLFSLIPSAISGLISAFK-NH_2_	1.54	(+ 1/ + 1)	0.44	6.19	91.59	121	1.15 E4	1.67	–3.69	–1.32	6.05
2143 (E4VP07)	Meucin-13, VAP-6	IFGAIAGLLKNIF-NH_2_	1.70	(+ 1/ + 1)	0.71	7.39	137.83	121	1.05 E4	1.57	–2.54	–1.83	5.64
2144 (E4VP50)	Meucin-18, VAP-9	FFGHLFKLATKIIPSLFQ	0.85	(0/ + 2)	0.54	8.55	119.72	261	1.27 E4	1.73	–3.19	–0.66	5.35
2159 (Q8MMJ7)	IsCT	ILGKIWEGIKSLF-NH_2_	0.77	(+ 1/ + 1)	0.77	9.86	117.44	125	9.98 E3	1.97	–0.88	–0.80	6.97
2160 (Q8MMJ7)	IsCT [WA6]	ILGKIAEGIKSLF-NH_2_	0.98	(+ 1/ + 1)	0.73	10.27	117.45	124	9.64 E3	2.01	1.14	–0.76	7.00
2161 (Q8MMJ7)	IsCT [WL6]	ILGKILEGIKSLF-NH_2_	1.13	(+ 1/ + 1)	0.76	10.06	115.79	124	9.90 E3	2.00	0.41	–1.00	6.67
2162	IsCT [EK7]	ILGKIWKGIKSLF-NH_2_	0.74	(+ 1/ + 3)	0.80	13.35	117.44	163	9.20 E3	2.00	–1.91	–0.90	7.04
2163	IsCT [WL6, SK11]	ILGKILKGIKKLF-NH_2_	0.86	(+ 1/ + 4)	0.85	14.33	118.38	170	9.16 E3	2.15	0.24	–0.93	6.01
2164	IsCT [EK7, GP8, SK11]	ILGKIWKPIKKLF-NH_2_	0.41	(+ 1/ + 4)	0.86	14.15	117.38	170	9.16 E3	2.21	–0.61	–0.66	6.25
3246	IsCT2f	IFGAIWNGIKS	0.74	(0/ + 1)	0.64	7.46	140.16	125	9.02 E3	1.75	–2.18	–0.74	7.02
3247	IsCT2	IFGAIWNGIKSLF-NH_2_	1.13	(+ 1/ + 1)	0.71	7.61	131.47	118	1.04 E4	1.66	–3.87	–1.23	5.72
3248	IsCTf	ILGKIWEGIKS	0.31	(0/ + 1)	0.71	8.76	127.30	134	8.50 E3	2.18	0.81	–0.23	14.7
3343	Mastoparan-L	INLKALAALAKKIL-NH_2_	1.15	(+ 1/ + 3)	0.39	11.34	69.04	151	1.04 E4	1.80	1.21	–0.96	7.07
3353	Hp1090, Um5	IFKAIWSGIKSLF-NH_2_	1.07	(+ 1/ + 2)	0.72	11.93	127.17	161	9.99 E3	1.77	–3.18	–0.98	5.59
3354	Hp1035	IFSAIGGFLKSIF-NH_2_	1.63	(+ 1/ + 1)	0.69	8.35	132.16	119	9.39 E3	1.65	–3.44	–1.53	5.34
3475	VmCT1	FLGALWNVAKSVF-NH_2_	1.20	(+ 1/ + 1)	0.57	7.13	139.04	119	8.33 E3	1.60	–3.20	–1.16	5.37
3476	VmCT2	FLSTLWNAAKSIF-NH_2_	0.82	(+ 1/ + 1)	0.60	8.22	135.35	117	8.56 E3	1.75	–3.39	–0.39	6.11
3477	VsCT1	FLKGIIDTVSNWL-NH_2_	0.76	(+ 1/0)	0.76	8.66	126.18	164	1.23 E4	1.87	–1.73	–0.23	5.32
3478	VsCT2	FLKGIIDTVSKLF-NH_2_	1.01	(+ 1/ + 1)	0.76	10.67	124.25	141	8.65 E3	1.97	–0.44	–0.37	5.35
3479	CT1-NDBP-5.17, UyCT1	GFWGKLWEGVKNAI-NH_2_	–0.05	(+ 1/ + 1)	0.69	8.84	52.41	140	1.32 E4	1.95	–1.01	–0.11	11.9
3480	CT1-NDBP-5.17, UyCT2	FWGKLWEGVKNAI-NH_2_	–0.02	(+ 1/ + 1)	0.74	9.21	116.41	131	8.55 E3	1.98	–1.02	–0.05	7.89
3481	CT3-NDBP-5.15, UyCT3, OcyC1, NDBP-5.7	ILSAIWSGIKSLF-NH_2_	1.39	(+ 1/ + 1)	0.66	8.00	131.62	116	1.01 E4	1.70	–3.47	–1.30	5.88
3482	CT5-NDBP-5.16, UyCT5	IWSAIWSGIKGLL-NH_2_	1.13	(+ 1/ + 1)	0.69	8.82	124.08	116	1.07 E4	1.66	–4.31	–1.58	5.53
3489	Pantinin-2	IFGAIWKGISSLL-NH_2_	1.42	(+ 1/ + 1)	0.71	9.82	118.77	142	1.09 E4	1.67	–3.59	–1.63	5.36
3490	Pantinin-1	GILGKLWEGFKSIV-NH_2_	0.67	(+ 1/ + 1)	0.69	9.53	54.71	137	1.16 E4	1.96	–0.49	–0.75	5.32
3491	Pantinin-3	FLSTIWNGIKSLL-NH_2_	0.93	(+ 1/ + 1)	0.70	8.99	131.34	119	9.21 E3	1.84	–3.46	–0.71	5.79
3599	CT1	GFWGSLWEGVKSVV-NH_2_	0.51	(+ 1/0)	0.61	9.08	63.20	121	9.12 E3	1.85	–1.88	–0.59	6.84
3615	CT2, Um3	GFWGKLWEGVKSAI-NH_2_	0.14	(+ 1/ + 1)	0.65	9.11	54.47	138	1.24 E4	1.95	–1.30	–0.34	16.0
3752	Mucroporin	LFGLIPSLIGGLVSAFK-NH_2_	1.61	(+ 1/ + 1)	0.58	5.44	142.18	110	1.10 E4	1.68	–3.15	–1.87	5.41
3753	Mucroporin-M1, Mucroporin [G3,11R, P6K, G10K]	LFRLIKSLIKRLVSAFK-NH_2_	0.79	(+ 1/ + 5)	0.74	13.85	134.29	196	1.39 E4	1.89	–0.03	0.70	5.93
3754	Imcroporin	FFSLLPSLIGGLVSAIK-NH_2_	1.59	(+ 1/ + 1)	0.58	7.11	132.72	116	1.10 E4	1.70	–3.03	–1.61	8.14
3884	1, AamAP1	FLFSLIPHAIGGLISAFK-NH_2_	1.43	(+ 1/ + 1)	0.43	5.95	89.48	119	1.13 E4	1.57	–3.77	–1.49	6.00
3885	2, AamAP2	FPFSLIPHAIGGLISAIK-NH_2_	1.22	(+ 1/ + 2)	0.39	6.68	85.75	125	1.11 E4	1.71	–1.94	–1.33	9.43
3886	AamAP1 [H8K]	FLFSLIPKAIGGLISAFK-NH_2_	1.39	(+ 1/ + 1)	0.49	7.95	76.71	137	1.12 E4	1.70	–2.95	–1.44	6.39
3935	Ctriporin	FLWGLIPGAISAVTSLIKK-NH_2_	1.16	(+ 1/ + 2)	0.45	5.87	93.98	83	1.28 E4	1.77	–2.33	–1.25	7.09
4297	TsAP-1	FLSLIPSLVGGSISAFK-NH_2_	1.32	(+ 1/ + 1)	0.47	4.95	139.47	111	1.11 E4	1.84	–2.34	–1.12	6.10
4298	TsAP-2	FLGMIPGLIGGLISAFK-NH_2_	1.54	(+ 1/ + 1)	0.59	4.94	146.06	109	9.88 E3	1.65	–3.32	–2.02	5.49
4299	TsAP-1 [S7K] [G10K] [G11K] [S12I] [S14K]	FLSLIPKLVKKIIKAFK-NH_2_	0.85	(+ 1/ + 5)	0.75	12.52	158.80	170	1.07 E4	2.06	0.90	–0.59	6.29
4300	TsAP-2 [G7K] [G10K] [G11K] [S14K]	FLGMIPKLIKKLIKAFK-NH_2_	0.74	(+ 1/ + 5)	0.77	12.59	161.49	179	1.01 E4	2.04	0.48	–0.75	5.39
4330	Peptide Hp1036	ILGKIWEGIKSIF-NH_2_	0.83	(+ 1/ + 1)	0.78	9.90	117.49	125	9.77 E3	1.95	–0.63	–0.80	5.81
4331	Peptide Hp1239	ILSYLWNGIKSIF-NH_2_	0.94	(+ 1/ + 1)	0.68	7.90	133.44	119	1.05 E4	1.66	–4.29	–0.90	5.35
4572	Kn2–7	FIKRIARLLRKIF-NH_2_	0.55	(+ 1/ + 5)	0.90	16.61	124.49	199	1.20 E4	1.84	0.27	1.8	6.08
7227	AamAP1 [S4K, H8K, G11,12 K, A16K]	FLFKLIPKAIKKLISKFK-NH_2_	0.51	(+ 1/ + 6)	0.60	12.06	60.99	174	1.11 E4	2.20	0.69	–0.19	5.59
8151	AaeAP1	FLFSLIPSVIAGLVSAIRN-NH_2_	1.58	(+ 1/ + 1)	0.45	5.68	89.36	135	1.40 E4	1.60	–2.31	–0.86	6.93
8152	AaeAP2	FLFSLIPSAIAGLVSAIRN-NH_2_	1.45	(+ 1/ + 1)	0.42	5.56	88.22	136	1.39 E4	1.61	–2.21	–0.74	7.32
8153	AaeAP1 [S4,8,15 K; A11K, N19K]	FLFKLIPKVIKGLVKAIRK-NH_2_	0.77	(+ 1/ + 6)	0.66	13.29	76.17	216	1.25 E4	2.01	1.66	–0.19	8.40
8154	AaeAP2 [S4,8,15 K; A11K, N19K]	FLFKLIPKAIKGLVKAIRK-NH_2_	0.64	(+ 1/ + 6)	0.64	13.18	75.43	214	1.24 E4	2.03	1.76	–0.07	8.98
8199	Stigmurin	FFSLIPSLVGGLISAFK-NH_2_	1.53	(+ 1/ + 1)	0.57	6.42	131.60	113	1.08 E4	1.69	–3.60	–1.50	6.15
8437	VpAmp1.0	LPFFLLSLIPSAISAIKKI-NH_2_	1.52	(+ 1/ + 2)	0.41	5.51	164.94	95	1.13 E4	1.79	–2.13	–1.45	7.23
8438	VpAmp1.1	FFLLSLIPSAISAIKKI-NH_2_	1.57	(+ 1/ + 2)	0.37	5.42	17.12	92	1.13 E4	1.73	–2.02	–1.33	6.77
8864	Hp1404	GILGKLWEGVKSIF-NH_2_	0.67	(+ 1/ + 1)	0.67	9.40	54.60	136	1.10 E4	1.96	–0.49	–0.75	5.60
9867	ToAP3, Im–4	FIGMIPGLIGGLISAIK-NH_2_	1.68	(+ 1/ + 1)	0.59	5.37	145.06	110	9.63 E3	1.65	–2.25	–2.14	6.05
9869	ToAP1	FIGMIPGLIGGLISAFK-NH_2_	1.58	(+ 1/ + 1)	0.59	4.85	148.15	107	9.67 E3	1.64	–3.07	–2.02	5.35
9870	OcyC2, NDBP-5.8	GILGKIWEGVKSLI-NH_2_	0.79	(+ 1/ + 1)	0.68	10.03	55.71	137	1.15 E4	1.97	0.33	–0.89	5.46
9945	Uy17	ILSAIWSGIKGLL-NH_2_	1.50	(+ 1/ + 1)	0.66	8.61	128.03	115	1.06 E4	1.68	–3.02	–1.78	5.33
9946	Uy192	FLSTIWNGIKGLL-NH_2_	0.96	(+ 1/ + 1)	0.70	9.14	128.68	117	8.94 E3	1.80	–3.58	–1.04	6.80
9947	Uy234	FPFLLSLIPSAISAIKRL-NH_2_	1.32	(+ 1/ + 2)	0.46	4.84	95.63	92	1.15 E4	1.79	–2.00	–0.74	7.11
9951	Um2	ISQSDAILSAIWSGIKSLF-NH_2_	0.83	(+ 1/0)	0.50	8.84	74.18	113	1.48 E4	1.89	–1.54	–0.13	23.2
9952	Um4	FFSALLSGIKSLF-NH_2_	1.49	(+ 1/ + 1)	0.65	8.09	130.51	121	7.79 E3	1.70	–3.82	–1.20	5.63
9953	UyCT3 [L2F,S3G], D1	IFGAIWSGIKSLF-NH_2_	1.36	(+ 1/ + 1)	0.67	7.46	131.27	117	9.63 E3	1.65	–4.16	–1.48	5.65
9954	Uy192 [G11S,L13F], D2	FLSTIWNGIKSLF-NH_2_	0.86	(+ 1/ + 1)	0.71	8.55	130.65	118	8.53 E3	1.82	–4.03	–0.56	5.36
9955	UyCT1 [E8K,G9P,N12K], D4	GFWGKLWKPVKKAI-NH_2_	–0.19	(+ 1/ + 4)	0.73	12.10	37.32	173	1.15 E4	2.17	–1.03	–0.21	20.1
9956	UyCT1 [W7L,N12K], D5	GFWGKLLEGVKKAI-NH_2_	0.25	(+ 1/ + 2)	0.70	9.31	40.87	121	1.23 E4	2.08	0.85	–0.37	16.6
9957	UyCT1 – 3 K, D11	GFWGKLWEGVKNAIKKK-NH_2_	–0.72	(+ 1/ + 4)	0.59	5.42	34.90	52	1.34 E4	2.47	1.96	0.88	5.80
10158	NDBP-5.5	IFSAIAGLLSNLL-NH_2_	1.93	(+ 1/0)	0.65	8.14	123.31	134	8.50 E3	1.56	–2.96	–1.81	5.45
10462	IsCT1 [I5,9A]	ILGKAWEGAKSLF-NH_2_	0.36	(+ 1/ + 1)	0.56	9.46	114.16	125	9.87 E3	2.04	0.07	–0.32	9.59
10463	IsCT1 [I5,9 V]	ILGKVWEGVKSLF-NH_2_	0.73	(+ 1/ + 1)	0.69	9.75	115.63	125	1.01 E4	1.99	–0.12	–0.67	7.21
10464	IsCT1 [I5,9L]	ILGKLWEGLKSLF-NH_2_	0.66	(+ 1/ + 1)	0.76	9.94	119.27	126	1.03 E4	2.02	–1.38	–0.80	8.02
10465	IsCT1 [K10E]	ILGKIWEGIESLF-NH_2_	0.80	(+ 1/–1)	0.76	9.26	133.65	177	1.13 E4	1.94	0.15	–0.70	7.12
10466	IsCT2 [F2L;I5,9A]	ILGAAWNGAKSLF-NH_2_	0.80	(+ 1/ + 1)	0.49	7.21	130.56	116	1.05 E4	1.73	–2.34	–0.90	7.37
10467	IsCT2 [F2L;I5,9 V]	ILGAVWNGVKSLF-NH_2_	1.16	(+ 1/ + 1)	0.62	7.50	131.89	116	1.08 E4	1.69	–2.54	–1.25	6.52
11211	MeuFSPL-2	FLFSLIPSAISGLINAFK-NH_2_	1.39	(+ 1/ + 1)	0.47	6.12	90.98	124	1.22 E4	1.68	–3.40	–1.14	5.87
11223	Spiniferin	ILGEIWKGIKDIL-NH_2_	0.70	(+ 1/0)	0.83	9.45	136.26	132	1.15 E4	2.02	1.04	–0.54	5.41
11224	Spiniferin [E4K,D11N]	ILGKIWKGIKNIL-NH_2_	0.66	(+ 1/ + 3)	0.84	13.35	118.37	170	1.03 E4	2.02	–0.80	–0.80	5.34
11270	Stigmurin [S7K,G10K], StigA6	FFSLIPKLVKGLISAFK-NH_2_	1.14	(+ 1/ + 3)	0.66	10.36	142.96	152	1.09 E4	1.85	–1.76	–0.99	6.26
11271	Stigmurin [S3,7 K; G10K], StigA16	FFKLIPKLVKGLISAFK-NH_2_	0.96	(+ 1/ + 4)	0.72	14.63	135.00	209	1.09 E4	1.93	–0.90	–0.86	7.52
12239	HAP-1 (1–19)	QKDDEEESRFFFNFIFSAE-NH_2_	–0.93	(+ 1/–4)	0.22	10.15	90.35	146	1.96 E4	2.94	7.81	3.28	8.67
12417	Stigmurin [G10,11 K; S14K], StigA25	FFSLIPSLVKKLIKAFK-NH_2_	0.94	(+ 1/ + 4)	0.70	9.91	167.31	132	1.08 E4	1.97	–0.78	–0.61	5.59
12418	Stigmurin [S3,6,14 K; G10,11 K], StigA31	FFKLIPKLVKKLIKAFK-NH_2_	0.57	(+ 1/ + 6)	0.80	15.39	148.69	222	1.08 E4	2.16	0.94	–0.35	6.37
12573	1, AamAP1[P7R; H8K]	FLFSLIRKAIGGLISAFK	1.23	(+ 1/ + 3)	0.51	8.08	88.82	217	1.33 E4	1.68	–2.59	–0.61	6.75
13647	AcrAP1, AP1–Z1	FLFSLIPHAISGLISAFK-NH_2_	1.41	(+ 1/ + 1)	0.43	6.19	90.11	121	1.16 E4	1.60	–3.65	–1.25	5.81
13648	AcrAP1 [S4K, H8K, S11K, S15K]	FLFKLIPKAIKGLIKAFK-NH_2_	0.85	(+ 1/ + 5)	0.64	14.16	78.24	211	1.06 E4	1.94	–0.25	–0.84	6.31
1349	AcrAP2	FLFSLIPNAISGLLSAFK-NH_2_	1.35	(+ 1/ + 1)	0.47	6.54	88.16	121	1.23 E4	1.68	–3.65	–1.14	5.55
13650	AcrAP2 [S4K, N8K, S11K, S15K]	FLFKLIPKAIKGLLKAFK-NH_2_	0.81	(+ 1/ + 5)	0.63	14.33	77.66	212	1.07 E4	1.95	–0.50	–0.84	5.58
14214	IsCT [G3K, E7K, G8NAla, S11K]	ILKKIWKXIKKLF-NH_2_^b^	0.40	(+ 1/ + 5)	0.91	17.56	120.76	208	9.54 E3	2.17	0.09	–0.30	−
14497	QnCs-BUAP	FFSLIPSLISGLI-NH_2_	2.00	(+ 1/0)	0.60	7.92	114.19	132	8.00 E3	1.65	–4.02	–2.01	7.38
14624	Marcin-18	FFGHLFKLATKIIPSLFR-NH_2_	0.80	(+ 1/ + 3)	0.59	10.81	115.48	176	1.24 E4	1.72	–2.96	–0.14	5.32
14625	Megicin-18	FFGALFKLATKIIPSLFR-NH_2_	1.07	(+ 1/ + 3)	0.58	10.78	116.52	180	1.26 E4	1.72	–2.96	–0.50	5.32
14626	VmCT1 [G3R]	FLRALWNVAKSVF-NH_2_	0.89	(+ 1/ + 2)	0.63	11.54	133.00	158	9.36 E3	1.62	–2.40	0.05	5.52
14627	VmCT1 [N7R]	FLGALWRVAKSVF-NH_2_	1.13	(+ 1/ + 2)	0.60	9.52	141.05	133	8.69 E3	1.59	–2.81	–0.53	5.33
14634	VmCT1 [S11R]	FLGALWNVAKRVF-NH_2_	0.92	(+ 1/ + 2)	0.63	7.86	136.92	105	8.93 E3	1.60	–2.52	–0.28	5.36
14635	VmCT1 [F1G]	GLGALWNVAKSVF-NH_2_	0.96	(+ 1/ + 1)	0.51	7.82	128.75	123	8.59 E3	1.67	–2.06	–1.01	7.77
14636	VmCT1 [V8P]	FLGALWNPAKSVF-NH_2_	0.76	(+ 1/ + 1)	0.57	6.85	139.33	118	8.12 E3	1.76	–2.82	–0.85	5.53
14637	VmCT1 [A9L]	FLGALWNVLKSVF-NH_2_	1.36	(+ 1/ + 1)	0.67	7.64	137.84	121	8.56 E3	1.59	–3.93	–1.40	5.42
14638	VmCT1 [A9F]	FLGALWNVFKSVF-NH_2_	1.28	(+ 1/ + 1)	0.68	7.23	137.40	121	8.42 E3	1.57	–4.95	–1.25	5.54
14639	VmCT1 [V12L]	FLGALWNVAKSLF-NH_2_	1.17	(+ 1/ + 1)	0.60	7.56	137.68	120	8.42 E3	1.60	–3.83	–1.23	5.69
14640	VmCT1 [V12Y]	FLGALWNVAKSYF-NH_2_	0.78	(+ 1/ + 1)	0.56	5.85	140.91	122	1.10 E4	1.58	–4.21	–0.84	5.74
15558	Eval418	LWGEIWNTVKGLI-NH_2_	0.50	(+ 1/0)	0.74	8.09	125.59	96	9.52 E3	1.85	–1.78	–0.66	5.37
15559	Eval418-FH2	LWGHIWNFVHGLI-NH_2_	0.85	(+ 1/0)	0.67	7.84	115.70	134	8.63 E3	1.39	–5.72	–1.32	5.33
15560	Eval418-FH3	LWHHIWNFVHGLI-NH_2_	0.63	(+ 1/0)	0.67	8.28	115.70	135	8.97 E3	1.32	–5.56	–0.89	7.60
15561	Eval418-FH4	LWHHIWNTVHHLI-NH_2_	0.15	(+ 1/0)	0.66	7.74	114.70	136	9.61 E3	1.32	–4.13	–0.04	6.53
15562	Eval418-FH5	LWHHIWHTVHHLI-NH_2_	0.17	(+ 1/0)	0.60	7.54	112.18	136	9.09 E3	1.25	–4.38	–0.19	6.30
16149	MK049518	FLGLLGSVLGSVLPSIFK-NH_2_	1.57	(+ 1/ + 1)	0.46	6.79	119.62	127	1.14 E4	1.75	–3.37	–1.70	5.36
16150	MK049518 [S7,11,15 K]	FLGLLGKVLGKVLPKIFK-NH_2_	1.06	(+ 1/ + 4)	0.57	11.76	113.76	176	1.15 E4	1.94	–0.79	–1.34	5.42
16151	MK049518 [G3,6,10 K; S7,11,15 K]	FLKLLKKVLKKVLPKIFK-NH_2_	0.47	(+ 1/ + 7)	0.62	15.71	125.63	240	1.19 E4	2.34	2.15	–0.26	10.9
16788	VmCT1 [F1K]	KLGALWNVAKSVF-NH_2_	0.69	(+ 1/ + 2)	0.49	11.37	95.58	160	8.31 E3	1.80	–1.08	–0.51	10.6
16789	VmCT1 [A9K]	FLGALWNVKKSVF-NH_2_	0.76	(+ 1/ + 2)	0.48	5.25	134.06	108	8.60 E3	1.81	–2.38	–0.60	5.58
16790	VmCT1 [F1K,V12K]	KLGALWNVAKSKF-NH_2_	0.06	(+ 1/ + 3)	0.43	9.35	80.68	126	8.43 E3	2.08	–0.16	0.22	37.4
16791	VmCT1 [G3K,N7K]	FLKALWKVAKSVF-NH_2_	0.90	(+ 1/ + 3)	0.66	14.20	134.77	151	8.20 E3	1.79	–1.65	–0.75	7.16
16792	VmCT1 [G3K,S11K]	FLKALWNVAKKVF-NH_2_	0.70	(+ 1/ + 3)	0.69	11.64	133.94	138	8.37 E3	1.79	–1.36	–0.50	6.22
16793	VmCT1 [N7K,S11K]	FLGALWKVAKKVF-NH_2_	0.93	(+ 1/ + 3)	0.66	10.77	137.16	180	7.53 E3	1.74	–1.77	–1.08	5.34
16794	VmCT1 [G3K,N7K,S11K]	FLKALWKVAKKVF-NH_2_	0.66	(+ 1/ + 4)	0.72	14.20	136.13	115	7.85 E3	1.87	–0.79	–0.58	6.77
17518	BmKn1	FIGAVAGLLSKIF-NH_2_	1.88	(+ 1/ + 1)	0.63	7.56	116.56	108	9.98 E3	1.58	–2.45	–2.01	5.45
17526	Im5	FLGSLFSIGSKLLPGVIKLFQRKKQ-NH_2_	0.33	(+ 1/ + 5)	0.432	8.182	114.61	125	1.77 E4	2.178	0.32	0.06	5.51
17527	Im6	FFFLPSLIGGLVSAIK-NH_2_	1.68	(+ 1/ + 1)	0.43	5.02	67.12	118	1.06 E4	1.63	–3.73	–1.80	7.20
18163	VpCT1	FWSTLLSIGKSLL-NH_2_	1.09	(+ 1/ + 1)	0.58	8.57	128.82	118	8.80 E3	1.86	–4.0	–0.96	7.06
18164	VpCT2	FWSTIWNAAKSLI-NH_2_	0.59	(+ 1/ + 1)	0.63	8.65	126.28	102	9.05 E3	1.74	–3.86	–0.34	11.3
18165	VpCT3	FLQGIIDTVGKWL-NH_2_	0.79	(+ 1/0)	0.75	7.37	117.28	102	1.18 E4	1.84	–1.69	–0.65	6.03
18166	VpCT3 [I6W]	FLQGIWDTVGKWL-NH_2_	0.37	(+ 1/0)	0.76	7.08	123.17	119	1.21 E4	1.82	–3.23	–0.45	5.88
18167	VpCT4	LWGALLGLGSTLLSKL-NH_2_	1.25	(+ 1/ + 1)	0.53	5.37	110.98	140	1.34 E4	1.78	–4.18	–1.65	5.67
18168	VpCTconsensus	FLSKIWDGVKSLL-NH_2_	0.66	(+ 1/0)	0.73	11.02	118.93	120	8.41 E3	2.01	–1.42	–0.25	5.87
1892	Ctri9594	GVVDTLKNLLMGLL-NH_2_	1.20	(+ 1/0)	0.55	9.90	83.02	141	1.41 E4	1.79	–0.09	–0.95	6.01
20289	IsCT [E7P]	ILGKIWPGIKSLF-NH_2_	0.92	(+ 1/ + 2)	0.67	10.45	111.51	161	8.77 E3	1.93	–2.45	–1.32	6.83
20290	IsCT [G3K,G8P]	ILKKIWEPIKSLF-NH_2_	0.41	(+ 1/ + 2)	0.82	13.39	117.82	172	1.02 E4	2.24	0.54	–0.23	14.3
20291	IsCT [I1A; G3,8 K; I5F]	ALKKFWEKIKSLF-NH_2_	–0.1	(+ 1/ + 3)	0.80	14.51	103.94	159	8.24 E3	2.36	0.74	0.58	31.9
20292	IsCT [I1A, I5F, E7P, G8K]	ALGKFWPKIKSLF-NH_2_	0.32	(+ 1/ + 3)	0.67	12.61	95.27	170	7.03 E3	2.10	–1.81	–0.44	12.1
20293	IsCT [G3K,E7K,I9K]	ILKKIWKGKKSLF-NH_2_	–0.17	(+ 1/ + 5)	0.64	14.77	117.33	169	9.78 E3	2.79	0.37	0.40	67.2
20294	IsCT [G3K, E7K, G8P, I9K]	ILKKIWKPKKSLF-NH_2_	–0.26	(+ 1/ + 5)	0.65	14.76	117.22	193	9.67 E3	2.86	0.81	0.47	28.1
21142	Hp1470	IFKAIWSGINRLF	0.82	(0/ + 2)	0.77	8.72	142.80	108	1.23 E4	1.69	–3.07	–0.01	5.33
21411	TtAP-2	IFGMIPGLIGGLISAFK-NH_2_	1.59	(+ 1/ + 1)	0.59	4.92	146.20	115	9.79 E3	1.64	–3.07	–2.02	5.37
21412	TtAP-3	FFSLIPSLIGGLVSAIK-NH_2_	1.64	(+ 1/ + 1)	0.59	7.16	131.28	135	1.08 E4	1.69	–2.78	–1.61	7.84

^a^Database of Antimicrobial Activity and Structure of Peptides, DBAASP and (Uniprot); ^b^X: Sarcosine; ^c^Values based on the estimates of Juretić et al. (2009)

This could suggest that the mechanism of action of ssAMPs is probably not the formation of aqueous and stable membrane pores, as melittin or magainins does (Jamasbi et al. 2016; Pino-Angeles et al. 2016). Rather, the available evidence indicates that peptides as short as Stigmurin (17 aa) or Ctriporin (19 aa) are quite stable in micellar systems (Daniele-Silva et al. 2021; Bandyopadhyay et al. 2014). On the other hand, there is experimental evidence supported by MD studies that longer peptides such as Pin2 (24-aa) form toroidal pores (Nomura et al. 2004; Velasco-Bolom and Garduño-Juárez 2022). It seems that the vast majority of ssAMPs could promote micellization phenomena in a dose-dependent manner. However, since there is the possibility that peptides as small as gramicidin (15 L- and D-aa) or aurein 1.2 (13 aa) being too short are also capable of forming aqueous pores by dimerization phenomena (Andersen et al. 2005; Lorenzón et al. 2013, 2016), we do not rule out this possibility for some amidated ssAMPs. In the case of the hypothetical pore formed by the dimerization of aurein 1.2, it has been shown that the presence of glycolipids determines the lifetime of the pore (Balatti et al. 2020). We are experimentally exploring the role of anionic lipids to have a clearer idea of the mechanism of action of the Uy234 peptide since we have evidence that C-terminal amidation provides helicity and rigidity which favors membrane insertion (Salazar-Hernandez et al. 2024). Thus, elucidation of the mechanisms of action of these ssAMPs also contributes to building a theoretical framework capable of enriching our knowledge for the design of increasingly better peptides based on structural modeling and AI algorithms. In this regard, the use of sequence-based molecular modeling algorithms utilizes the amino acid composition, the presence of some consensus motifs, and certain physicochemical properties such as hydrophobicity to generate structural information not previously considered in the study of the bioactivity of these peptides (Wang et al. 2022). Nowadays, work that could take months or years is accelerated, thanks to these in silico technologies, which integrate extensive databases in terms of sequence, structure, and physicochemical properties (Aguilera-Puga et al. 2024). These strategies are being consolidated in the form of several on-line servers. One of the first, AntiBP and AntiBP2, provides the user with preliminary information on the possible bioactivity of a peptide by entering an amino acid sequence (Lata et al. 2007, 2010). A more extensive list of these predictors based on machine learning algorithms has been published elsewhere (Aguilera-Puga et al. 2024). On the other hand, a current challenge is also to predict the possible toxic effects that could result from a given sequence or structure. For this purpose, it has not been easy to estimate or calculate the hemolytic index of AMPs; however, some important efforts have been made to do so (Juretić et al 2009; Robles-Loaiza et al. 2022; Yang and Xu 2024), although traditionally experimental work has been especially useful for this purpose.

## Some Inspiring Stories: ssAMPs as Potential New Drugs

The use of AMPs found in the venoms of some animals has been, since the discovery of melittin, a constant paradigm for the development of new antibiotics. Numerous analogs of melittin have been designed and these exhibit important antimicrobial activity against pathogenic bacterial strains. Likewise, it has been shown that these derivatives of melittin, present in bee venom, have low hemolytic activity, which constitutes one of the main attributes that must be considered to approve a new drug (Akbarzadeh-Khiavi et al. 2022). However, to date, none of these melittin derivatives have been approved for clinical use. More recently, Rad et al. (2023) have obtained new analogs of melittin through in silico design strategies, molecular modeling, and dynamic simulations with encouraging results for the development of novel antibiotics against key pathogens in the field of AMR: *S. aureus*, *A. baumannii*, *E. faecalis*, *P. pneumoniae*, for example. The venoms of some species of snakes are one step ahead since several drugs and diagnostic tools have been approved based on some toxins and peptides derived from those present in their venoms (Pérez-Peinado et al. 2020). Indeed, some of them, such as Captopril (Capoten®) from *Bothrops jararaca* or Eptifibatide (Integrilin®) from *Sistrurus miliarius barbouri*, are already on the market or are in clinical trials for the treatment of hypertension and acute coronary syndromes, respectively. Others, such as Crotoxin I from *Crotalus durissus terrificus*, are in the preclinical phase for the treatment of cancer. This information can be consulted in the U.S. National Library of Medicine (https://clinicaltrials.gov/ct2/home).

The great therapeutic potential that ssAMPs from scorpion venoms have shown is a gold mine for the discovery of new drugs against ESKAPE pathogens (*Enterococcus faecium*, *S. aureus*, *K. pneumoniae*, *A. baumannii*, *P. aeruginosa*, and *Enterobacter* species), which causes nosocomial infections and high degree of morbidity/mortality and multidrug resistance (Santajit and Indrawattana 2016; De Oliveira et al. 2020). However, clinical or preclinical studies with ssAMPs are still scarce, in part due to the difficulty of obtaining appreciable quantities of scorpion venom (*T. fuhrmanni* produces, for example, only 0.56 ± 0.27 mg of venom per milking, Gómez-Cardona et al. 2002). However, the use of chemical synthesis or heterologous expression of the coding genes for these biomolecules is an attractive strategy to overcome this challenge (Cid-Uribe et al. 2020). In any case, once the scorpion venom has been obtained, it is necessary to purify the peptides of interest using conventional HPLC and mass spectrometric tools (Olamendi-Portugal et al. 2016).

Here, we will name just some works of clinical interest: (1) VmCT1 analogs from the venom of *V. mexicanus* have shown to be quite effective against ESKAPE pathogens and some fungi (Pedron et al. 2019a,b); (2) several analogs derived from BmKn2, isolated from the scorpion *M. martensii*, show inhibitory activity against *S. aureus* MRSA and, by topical application, it protects mice from bacterial infections with low hemolytic activity (Cao et al. 2012a, b); (3) Stigmurin and TsAP-2, isolated from the venom of the scorpion *T. stigmurus*, are capable of reducing the migration of leukocytes, regulating the levels of the cytokine TNF-α42 levels, reducing inflammation in the lung and cecum of septic animals, in addition to possess excellent antibacterial activities against *S. aureus* and *E. faecalis* (Daniele-Silva et al. 2016); (4) AcrAP1a and AcrAP2a are two analogs of AcrAP1 and AcrAP2, respectively, found in the venom of the scorpion *A. crassicauda*, and with excellent activity against *S. aureus*, *C. albicans*, and particularly *E. coli*, in addition to showing a potent modulatory effect on cancer cell growth (Du et al. 2014); (5) the mature peptide NDBP-5.5 from *Hadrurus gertschi* venom was evaluated against *Mycobacterium abscessus* subsp. *massiliense* and it contributes to reducing the bacterial load with anti-inflammatory effects in the lungs of mice (Trentini et al. 2017).

Those studies, still scarce but notable, confirm the vast therapeutic potential that ssAMPs have against pathogenic bacteria and fungi, in addition to the fact that their activities are relevant even to inhibit the proliferation of some viruses. These include hepatitis-B, influenza SARS-CoV, and H5N1 viruses which are sensitive to Mucroporin-M1 derivatives (Li et al. 2011; Zhao et al. 2012), Eval418 and Eval418-FH5 peptides against HSV-1 virus (Zeng et al. 2018), or Kn2-7 derivatives against the HIV-1 (Chen et al. 2012).

## Concluding Remarks

The powerful antimicrobial activity that has been reported for ssAMPs discovered in scorpion venoms is particularly linked to their physicochemical, structural, and thermodynamic properties. The presence of a central Pro in the Stigmurin-like group, a Trp residue in the pantinin-like group, the almost ubiquitous C-terminal amidation, and their high hydrophobicities toward the N-terminal end seem typical characteristics of the ssAMPs. In addition, we found that both the electrostatic potential and the hydrophobic moment make them more effective, and both parameters are especially important in longer members (17 or 18-aa) such as TsAP-1, Stigmurin derivatives, Marcin-18, or Megicin-18 but not in the shorter ones (13-aa). In addition, we found that longer peptides are generally more flexible but this feature reduces their bioactivity in terms of MIC, which would reinforce the idea that for their effectiveness, ssAMPs must remain ordered during the interaction with the lipid membrane. On the other hand, a common problem we encountered was the fact that ssAMPs especially active against pathogenic bacteria are also very hemolytic, suggesting a delicate balance between each parameter and the need to evaluate each case separately. In any case, each interaction must be studied in its own context since there does not seem to be a general correlation that would allow establishing an a priori predictability criterion to anticipate the bioactivity of these peptides.

To this end, properly characterizing each physicochemical parameter is just one part of the story. Considering the main characteristics of the lipid composition present in target cells could surely serve as a basis for optimizing new derivatives capable of attacking the growing presence of microbial pathogens resistant to conventional antimicrobial drugs that we have used for more than a century. The intensive in silico work, the use of diverse prediction tools to quantify the physicochemical, structural, and thermodynamic parameters, both of peptides and lipids, as well as the increasingly sophisticated molecular dynamics simulations, will contribute soon to establishing a link very close between the experimental results obtained in our laboratories and the validation of the theoretical performance that these potential new drugs could have.

## Note Added in Proof

The antimicrobial potential of the short peptides from scorpion venoms has also been recently reviewed by Panayi et al. (2024), which confirms the great relevance in the study and prediction of specific physicochemical parameters that are discussed in detail in the present work.

## Data Availability

MIC, hemolysis, physicochemical, and thermodynamic parameters of ssAMPs and some synthetic analogs are incorporated in the following repository: https://github.com/dballeza/ssAMPs
